# Biogenic silver nanoparticle-loaded injectable thermosensitive chitosan hydrogels accelerate repair of non-infected burn wounds

**DOI:** 10.3389/fbioe.2026.1990402

**Published:** 2026-10-28

**Authors:** Anam Ahsan, Qurat-ul-Ain Aslam, George Fu Gao, Wen-Xia Tian

**Affiliations:** 1 College of Animal Science & Veterinary Medicine, Shanxi Agricultural University, Taigu, China; 2 Drug Testing Laboratory, Faisalabad, Punjab, Pakistan; 3 Institute of Microbiology, Chinese Academy of Sciences, Beijing, China

**Keywords:** Aloe barbadensis, burn wound healing, chitosan hydrogel, injectable hydrogel, phytogenic nanoparticles, Ricinus communis, silver nanoparticles, thermosensitive hydrogel

## Abstract

Burn wounds are susceptible to microbial contamination, inflammation, fluid loss and delayed repair, highlighting the need for local formulations that conform to wound beds and support healing. Silver nanoparticles (AgNPs) have broad antibacterial potential, but direct topical application can be limited by aggregation, burst exposure and local toxicity. Here, phytogenic AgNPs previously synthesized from *Ricinus communis* and *Aloe barbadensis* leaf extracts were incorporated into injectable thermosensitive chitosan/glycerol-2-phosphate hydrogels. The formulations were designated Ch-HI, Ch/Rc-AgNP-HI and Ch/Ab-AgNP-HI and evaluated for physicochemical properties, sol-gel transition, swelling, AgNP content and entrapment efficiency, apparent release of Ag-containing species, erosion, morphology, thermal behavior, antibacterial activity, cytocompatibility and burn wound repair. The AgNP-loaded hydrogels showed near-physiological pH, rapid gelation near body temperature, porous morphology, apparent Ag-containing species release over 30 h and gradual matrix erosion over 21 days. The formulations demonstrated *in vitro* antibacterial activity against *Escherichia coli* and *Staphylococcus aureus* and maintained HFF-1 fibroblast viability above the 70% cytotoxicity threshold across 1–50 μg/mL. In a female BALB/c mouse second-degree burn wound model, daily administration of 50 µL formulation for three consecutive days promoted wound closure; Ch/Rc-AgNP-HI and Ch/Ab-AgNP-HI achieved 99.34% ± 0.4% and 98.75% ± 0.6% wound contraction by day 14 and complete closure by day 21. Microbial surveillance showed no detectable bacterial growth in any group, indicating a non-infected burn wound repair model. Histological and biochemical analyses further showed improved re-epithelialization, collagen organization, hydroxyproline deposition and dry matter recovery. These findings support phytogenic AgNP-loaded injectable chitosan/β-GP hydrogels as promising platforms for non-infected burn wound repair, with antibacterial potential demonstrated *in vitro*.

## 1 Introduction

Burn injuries remain a major clinical and public health challenge because thermal trauma disrupts the epidermal and dermal barrier, exposes underlying tissue to microbial colonization, and initiates a complex inflammatory response that can delay repair (Wang et al., 2018; Ivanko et al., 2024). Recent public-health analyses continue to highlight burns as a substantial global cause of mortality and morbidity, with approximately 180,000 burn-related deaths reported annually and the greatest burden occurring in low- and middle-income countries (Ivanko et al., 2024). Non-fatal burns also impose a substantial burden through prolonged hospitalization, pain, disability, disfigurement, scarring and psychological or social consequences (Peck and Toppi, 2019). Effective burn wound management therefore requires more than passive wound coverage; an ideal local therapy should protect the wound surface, reduce fluid loss, limit microbial contamination, maintain a moist microenvironment and support tissue regeneration (Markiewicz-Gospodarek et al., 2022).

Infection control is particularly important in burn wounds because loss of the skin barrier creates a nutrient-rich and immunologically vulnerable surface. Bacterial colonization can intensify inflammation, impair re-epithelialization, delay extracellular matrix deposition and increase the risk of systemic complications. Conventional burn dressings and topical antimicrobial preparations have improved clinical care, but important limitations remain (Wang et al., 2018). Gauze-based or poorly conformable dressings can adhere to the wound surface and disrupt newly formed epithelium during removal, while repeated dressing changes can increase pain and create opportunities for secondary contamination. Topical antimicrobial agents may also produce short-lived exposure, uneven distribution within irregular wounds or host-cell toxicity when local concentrations are poorly controlled (Goh et al., 2024; Li et al., 2025; Wang et al., 2025). These limitations motivate the development of advanced wound biomaterials that combine conformability, sustained antimicrobial delivery and tissue-supportive properties.

Hydrogels are attractive burn wound-care platforms because their hydrated three-dimensional polymer networks can absorb exudate, maintain moisture, reduce mechanical irritation and serve as local reservoirs for therapeutic agents (Hoffman, 2012; El-Sherbiny and Yacoub, 2013; Wang et al., 2018; Goh et al., 2024; Li et al., 2025). Among hydrogel systems, injectable thermosensitive hydrogels are especially valuable for irregular or difficult-to-dress wounds. These formulations can be handled as low-viscosity sols and then undergo sol-gel transition at or near physiological temperature, forming an *in situ* depot that conforms closely to the wound bed and improves local retention (Yu and Ding, 2008; Hoffman, 2012; El-Sherbiny and Yacoub, 2013; Kim et al., 2018; Ahsan et al., 2020). This sol-to-gel behavior is relevant for burn care because the formulation can be delivered into the wound surface without aggressive manipulation and can remain localized during the early repair period.

Chitosan is a favorable polymer for wound-healing hydrogels due to its biodegradability, biocompatibility, mucoadhesive character, hemostatic activity and intrinsic antimicrobial properties (Li et al., 2025; Nicolae-Maranciuc and Chicea, 2025; Wang et al., 2025). As a deacetylated derivative of chitin, chitosan contains protonatable amine groups that contribute to pH-responsive swelling and interactions with negatively charged biological surfaces. These properties can support wound repair by maintaining hydration, promoting local residence and providing a matrix that may facilitate cellular migration and extracellular matrix deposition. Our previous work also demonstrated that thermosensitive chitosan-based injectable hydrogels can act as depots for sustained delivery of bioactive agents (Ahsan et al., 2020).

Silver-based antimicrobial materials remain widely investigated for wound care because silver ions and silver nanomaterials can interact with bacterial membranes, proteins, enzymes and nucleic acids, resulting in broad antibacterial effects against Gram-negative and Gram-positive organisms (Huang et al., 2017; Díez-Pascual, 2018; Salleh et al., 2020; Alven and Aderibigbe, 2024). Silver nanoparticles (AgNPs) are particularly attractive because their nanoscale size and high surface area can enhance antimicrobial interactions. However, direct use of AgNPs may be limited by aggregation, burst release, uncontrolled local exposure and dose-dependent host-cell toxicity (Salleh et al., 2020; Haidari et al., 2021a; Haidari et al., 2021b; Pangli et al., 2021; Nicolae-Maranciuc and Chicea, 2025). Incorporating AgNPs into polymeric hydrogels provides a rational strategy to localize the particles, modulate AgNP/Ag + release, maintain antimicrobial activity and reduce uncontrolled exposure (Liu et al., 2020; Haidari et al., 2021a; Haidari et al., 2021b; Pangli et al., 2021; He et al., 2025; Nicolae-Maranciuc and Chicea, 2025).

Green synthesis offers an additional advantage for AgNP development because plant extracts contain phenolics, flavonoids, polysaccharides and other phytochemicals that can reduce silver ions and stabilize the resulting nanoparticles. The Rc-AgNPs and Ab-AgNPs used in the present study were previously synthesized using *R. communis* and *A. barbadensis* aqueous leaf extracts and characterized for physicochemical properties and biomedical potential (Ahsan et al., 2025). The current manuscript builds directly on that prior AgNP synthesis and *in vitro* characterization work. Therefore, the novelty of the present study does not lie in AgNP synthesis alone, but in incorporating two previously characterized phytogenic AgNP systems into injectable chitosan/glycerol-2-phosphate (β-GP) thermosensitive hydrogel depots and evaluating them through an integrated formulation-to-function workflow. This included hydrogel physicochemical characterization, *in vitro* antibacterial activity, short-term fibroblast compatibility and non-infected burn wound repair with microbial surveillance. Using two botanically distinct plant sources also allowed comparison of hydrogel systems containing AgNPs generated under different phytochemical capping conditions, while avoiding unsupported assumptions that one plant-derived AgNP system is inherently superior to the other.

Accordingly, this study aimed to prepare *R. communis*-derived AgNP-loaded chitosan hydrogel injection (Ch/Rc-AgNP-HI) and *A. barbadensis*-derived AgNP-loaded chitosan hydrogel injection (Ch/Ab-AgNP-HI) as injectable chitosan/β-GP thermosensitive hydrogel platforms for local burn wound repair. The study was designed as a formulation-to-function evaluation: first confirming hydrogel formation, pH, optical transition, network integrity, rheology, swelling, AgNP content, entrapment efficiency, apparent release of Ag-containing species and erosion; then assessing morphology, thermal behavior, *in vitro* antibacterial activity and short-term fibroblast compatibility; and finally evaluating macroscopic wound closure, microbial surveillance, histology, collagen organization, hydroxyproline content and dry matter recovery in a non-infected mouse burn wound model. We hypothesized that chitosan/β-GP thermosensitive hydrogels would enable local administration, *in situ* gelation, apparent release of Ag-associated species, preserved *in vitro* antibacterial activity, acceptable short-term fibroblast compatibility and improved repair outcomes in a non-infected burn wound model.

## 2 Materials and methods

### 2.1 Materials

Chitosan (molecular weight approximately 600 kDa; degree of deacetylation 86%) was purchased from Sigma-Aldrich (United States of America). Glycerol-2-phosphate (β-GP) was purchased from Merck (Germany). *Ricinus communis*-derived silver nanoparticles (Rc-AgNPs) and *Aloe barbadensis-*derived silver nanoparticles (Ab-AgNPs) were synthesized, lyophilized and stored as described previously (Ahsan et al., 2025). Trypsin was obtained from Thermo Fisher Scientific (United States of America). Dialysis membranes (molecular weight cut-off 3.5 kDa) were obtained from Shanghai GeneRay Biotech Co., Ltd. (Shanghai, China). *Escherichia coli* ATCC 25922 and *Staphylococcus aureus* ATCC 25923 were obtained from the Department of Microbiology, Shanxi Agricultural University. Gentamicin was used as the positive antibacterial control. All other reagents were of analytical grade and used without further purification.

### 2.2 Preparation of AgNP-loaded injectable thermosensitive chitosan hydrogels

A 2.5% (w/v) chitosan solution was prepared by dissolving chitosan powder in 0.1 M lactic acid at room temperature under continuous stirring until complete dissolution. For AgNP-loaded formulations, Rc-AgNPs or Ab-AgNPs (2.5 mg) were dispersed in 5 mL double-distilled water by sonication for 2 h, allowed to stand overnight, and further sonicated for 1 h to obtain homogeneous AgNP suspensions. Each AgNP suspension was then incorporated into the chitosan solution under continuous stirring, and the formulation volume was adjusted to 9 mL with 0.1 M lactic acid where required. Glycerol-2-phosphate (β-GP) solution (1 mL) was then added dropwise under continuous stirring for 10 min to obtain injectable thermosensitive hydrogel sols with a final volume of 10 mL. The blank chitosan hydrogel injection (Ch-HI) was prepared using the same method without AgNPs. The AgNP-loaded hydrogel formulations were designated *R. communis* AgNP-loaded chitosan hydrogel injection (Ch/Rc-AgNP-HI) and *A. barbadensis* AgNP-loaded chitosan hydrogel injection (Ch/Ab-AgNP-HI). Gelation was induced by incubation at 37 °C (Figure 1). All formulations were prepared in triplicate, and freeze-dried samples were stored in a desiccator until further characterization (Ahsan and Farooq, 2019; Ahsan et al., 2020).

**FIGURE 1:**
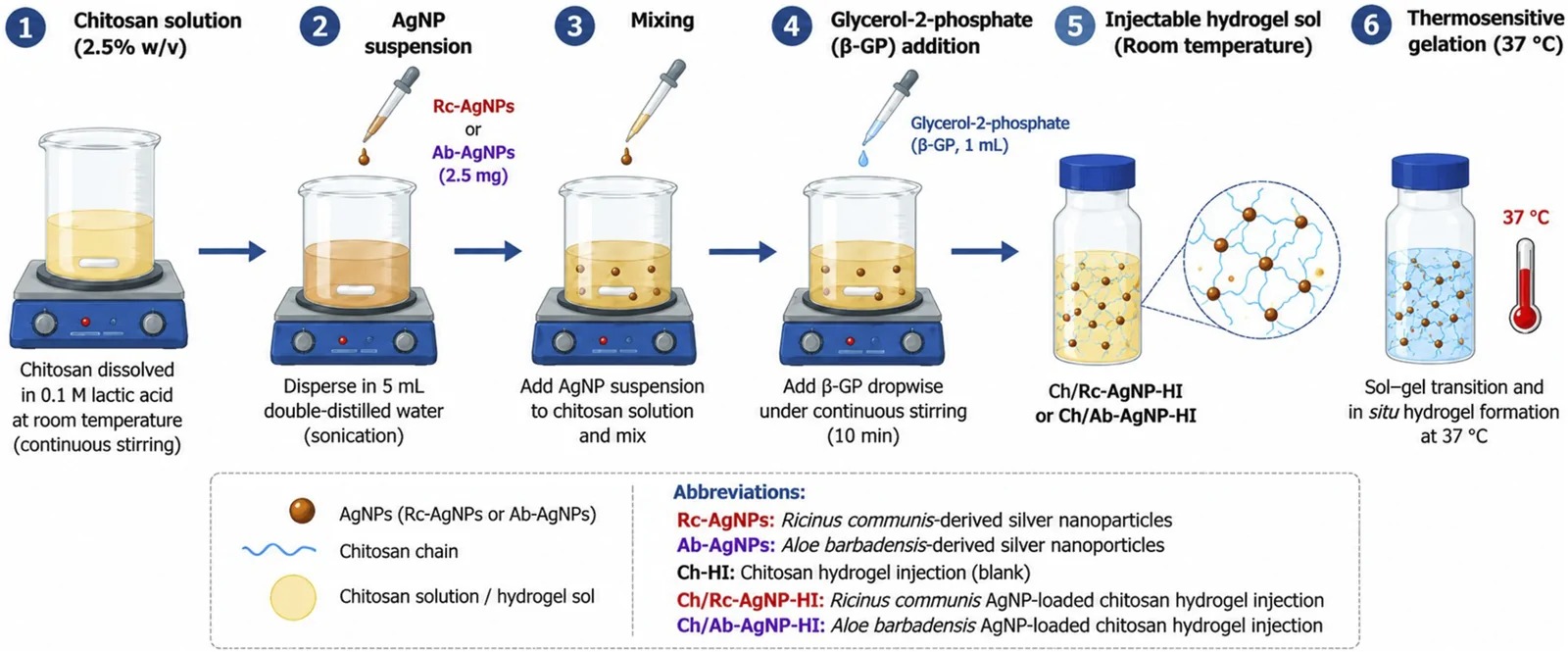
Schematic preparation of AgNP-loaded injectable thermosensitive chitosan hydrogels. A 2.5% (w/v) chitosan solution was prepared in 0.1 M lactic acid at room temperature. Rc-AgNPs or Ab-AgNPs were dispersed in double-distilled water by sonication and mixed with the chitosan solution. Glycerol-2-phosphate (β-GP) was then added dropwise under continuous stirring to neutralize the chitosan solution and obtain injectable thermosensitive hydrogel sols. Gelation was induced at 37 °C to form *in situ* hydrogel networks.

### 2.3 pH measurements

The pH of each hydrogel formulation was measured using a calibrated digital pH meter. Measurements were performed for the freshly prepared sol formulations and again after gelation to determine whether the sol-gel transition produced any substantial pH shift. All measurements were performed in triplicate at room temperature unless otherwise specified.

### 2.4 Visual transparency

The visual transparency of the prepared hydrogel injections was assessed over the temperature range relevant to handling and administration. Briefly, freshly prepared Ch-HI, Ch/Rc-AgNP-HI and Ch/Ab-AgNP-HI sols were inspected at room temperature and after incubation at 37 °C. The appearance of the formulations was recorded photographically to document the transition from a transparent or slightly opalescent injectable sol to a turbid gel following temperature-induced gelation.

### 2.5 Optical transparency as a function of temperature

Optical transparency was evaluated using a UV-visible spectrophotometer at 450 nm under variable temperature conditions. Double-distilled water was used as the 100% transmittance reference. Hydrogel sols were transferred into disposable cuvettes and placed in a digital water bath. The temperature increased stepwise, and the samples were allowed to equilibrate at each selected temperature for approximately 5 min before spectrophotometric measurement. The decrease in transmittance with increasing temperature was used as an optical indicator of thermosensitive sol-gel transition (Khan et al., 2018; Xue et al., 2020).

### 2.6 Sol-gel transition behavior

#### 2.6.1 Gelation temperature

Gelation temperature was evaluated using the vial inversion method. Freshly prepared hydrogel sols were placed in glass vials and heated gradually while being gently shaken and inverted at defined intervals. Gelation was considered complete when the formulation no longer flowed upon inversion of the vial. The temperature at which flow ceased was recorded as the gelation temperature. Measurements were performed for the AgNP-loaded hydrogel formulations in triplicate.

#### 2.6.2 Gelation time

Gelation time was measured using the test-tube inversion method. Briefly, 3 mL of freshly prepared hydrogel sol was transferred into a glass vial and incubated in a 37 °C water bath. At 1 min intervals, the vial was removed and inverted at a 90° angle for 1 min to observe whether flow persisted. The incipient gelation time was defined as the time at which visible solution flow stopped (Ghasemi Tahrir et al., 2016; Khan et al., 2018; Ahmad et al., 2019).

### 2.7 Sol-gel fraction

Sol-gel fraction analysis was performed to determine the soluble fraction and stable gel fraction of the hydrogel networks. Dried hydrogel discs were weighed before extraction and immersed in boiling water at 100 °C for 4 h to remove unreacted or soluble polymeric components. After extraction, the discs were removed from the water bath, dried at room temperature for 24 h and then incubated at 37 °C until further weighing. Gel fraction and sol fraction were calculated using Equations 1, 2, respectively:

$$Gel\;fraction\;\left(\%\right)=\left(\frac{W_{y}}{W_{x\;}}\right)\times 100$$ (1)

Sol fraction %=100−Gel fraction (2)

where W_x_ represents the initial dry hydrogel weight and W_y_ represents the dry weight after extraction. (Zaragoza et al., 2018; Alinejad et al., 2019; Kocak et al., 2020).

### 2.8 Texture analysis

Texture analysis was performed using a texture analyzer (XT Plus, Stable Micro Systems Ltd., UK) to assess the mechanical resistance of the hydrogel formulations after gelation. Hydrogel samples were allowed to undergo sol-gel transition before testing. A 10 mm Delrin probe was inserted into each gel at a speed of 1 mm/s to a depth of 5 mm. The maximum force recorded during probe insertion was taken as gel strength (N). Three readings were obtained for each formulation, and results were expressed as mean ± SD (Zaragoza et al., 2018; Alinejad et al., 2019; Kocak et al., 2020).

### 2.9 Rheological measurement

Rheological analysis was performed using a TA-AR 2000 rheometer (TA Instruments, United States of America) equipped with a 40 mm, 2° tapered geometry. Oscillatory measurements were conducted at a constant frequency of 1 Hz. During the temperature sweep, storage modulus (G′) and loss modulus (G″) were recorded as the temperature increased to evaluate thermosensitive sol-gel transition. The incipient gel point was defined as the crossover of G′ and G″, corresponding to tan delta (G″/G′) = 1. Time sweep analysis was then performed at 37 °C for 30 min to examine time-dependent strengthening of the gel network after exposure to physiological temperature (Zaragoza et al., 2018; Alinejad et al., 2019; Kocak et al., 2020).

### 2.10 Swelling index measurement

Swelling studies were performed to evaluate the water uptake behavior of the AgNP-loaded hydrogels under different temperature and pH conditions. Freeze-dried hydrogel samples were accurately weighed and immersed in buffered solutions at pH 1.2 or pH 7.4 at 25 or 37 °C under horizontal shaking. At predetermined time points, samples were removed from the swelling medium, gently blotted with filter paper to remove excess surface liquid and weighed. Swelling index (SI) was calculated using Equation 3:

$$SI=\frac{\left(W_{s}-W_{d}\right)}{W_{d}}$$ (3)

where Ws is the swollen hydrogel weight and Wd is the initial dry hydrogel weight. Measurements were performed in triplicate (Rehmani et al., 2017; Chee et al., 2018; Li et al., 2018).

### 2.11 AgNP content and entrapment efficiency

AgNP content and entrapment efficiency were determined by extracting AgNPs from weighed freeze-dried hydrogel samples. Briefly, hydrogel samples were crushed completely using a sterile pestle and mortar, dispersed in PBS and stirred continuously at 37 °C for 24 h to release incorporated AgNP-associated material from the hydrogel matrix. The dispersions were centrifuged at 3000 rpm, and the supernatant was collected and filtered through a 0.45 µm membrane. AgNP-associated content was quantified using UV–visible spectroscopy at the characteristic surface plasmon resonance absorbance region of the AgNPs, approximately 400–450 nm, using calibration curves prepared for the corresponding AgNPs.

AgNP content was expressed as mg AgNPs per 10 mL formulation, while entrapment efficiency was calculated as the percentage of the initially added AgNPs retained within the hydrogel formulation. AgNP content and entrapment efficiency were calculated using Equations 4, 5, respectively:

$$\text{AgNP content }\left(\text{mg}/10\text{ mL}\right)=\text{measured AgNP amount recovered}\text{from hydrogel formulation}$$ (4)

$$\text{Entrapment efficiency }\left(\%\right)=\left(\text{measured AgNP amount recovered}\text{from hydrogel formulation }/\text{ theoretical}\text{ AgNP amount added during}\text{formulation preparation}\right)\times 100$$ (5)

where the theoretical AgNP amount was 2.5 mg per 10 mL formulation. All measurements were performed in triplicate (Rehmani et al., 2017; Chee et al., 2018).

### 2.12 *In vitro* apparent release of Ag-containing species

*In vitro* release was evaluated using the dialysis membrane method to estimate the apparent release of diffusible Ag-containing species from the AgNP-loaded hydrogel formulations. Lyophilized hydrogel samples (2 mg) were placed in 5 mL PBS at pH 1.2 or pH 7.4 and transferred into dialysis bags with a molecular weight cut-off of 3.5 kDa. Both ends of the dialysis bags were securely sealed, and the bags were immersed in beakers containing 500 mL of the corresponding release medium. The release medium was maintained at 37 °C ± 1 °C with shaking at 100 rpm. At predetermined time points over 30 h, 3 mL aliquots were withdrawn from the external release medium and replaced immediately with an equal volume of freshly prepared PBS to maintain sink conditions.

The amount of released Ag-associated material was quantified by UV–visible spectroscopy at the characteristic surface plasmon resonance absorbance region of the AgNPs, approximately 400–450 nm, using calibration curves generated under the corresponding medium conditions. Cumulative apparent release (%) was calculated using Equation 6:

$$\text{Cumulative apparent release }\left(\%\right)=\left(\text{amount of}\text{ Ag}‐\text{ associated}\text{material detected in the release}\text{ medium}/\text{initial AgNP amount}\text{loaded in the hydrogel}\right)\times 100$$ (6)

Because the 3.5 kDa dialysis membrane is not expected to permit passage of intact AgNPs, the detected signal in the external release medium was interpreted as apparent release of diffusible Ag-containing species rather than confirmed release of intact nanoparticles. The release study was performed in triplicate for both formulations and both pH conditions (Rehmani et al., 2017; Chee et al., 2018; Li et al., 2018).

### 2.13 *In vitro* erosion

The *in vitro* erosion behavior of the hydrogels was evaluated by monitoring weight loss during incubation in PBS. Briefly, 2 mL of freshly prepared Ch/Rc-AgNP-HI or Ch/Ab-AgNP-HI sol was transferred into 15 mL tubes and incubated at 37 °C to allow complete gel formation. After gelation, 10 mL PBS was added to each tube, and the samples were maintained at 37 °C for predetermined time points of 3, 7, 14 and 21 days. At each time point, hydrogel samples were removed from the medium, gently blotted to remove excess surface PBS and weighed to determine the remaining hydrogel mass. The PBS medium was replaced every 3 days, and the experiment was performed in triplicate (Javaid et al., 2018; Li et al., 2018). The remaining hydrogel weight was calculated using Equation 7:

$$Remaining\;weight\;percent\;\left(\%\right)=\left(W_{t}/W_{o}\right)\times 100$$ (7)

where W0 is the initial hydrogel weight and Wt is the hydrogel weight after incubation in PBS for the specified time interval.

### 2.14 Morphological and thermal characterization

#### 2.14.1 Scanning electron microscopy

The internal microstructure of the blank and AgNP-loaded chitosan hydrogel injections was examined using scanning electron microscopy (SEM). Ch-HI, Ch/Rc-AgNP-HI and Ch/Ab-AgNP-HI samples were frozen at −80 °C for 48 h and then lyophilized for 24 h to preserve the hydrogel network architecture. The freeze-dried hydrogels were fractured in liquid nitrogen to expose internal cross-sections. Samples were mounted on SEM stubs, sputter-coated with gold using an Emitech K575 sputter coater and examined using a scanning electron microscope (JSM-5600, JEOL, Tokyo, Japan) at an acceleration voltage of 10 kV. The coating/imaging current was 20 mA, as recorded in the original method.

#### 2.14.2 Thermogravimetric analysis

Thermogravimetric analysis (TGA) was performed to evaluate the thermal degradation behavior of the hydrogel formulations. Dried hydrogel samples were crushed before analysis, and approximately 0.5–5 mg of each sample was placed in an open 100 µL platinum pan connected to the microbalance. Samples were analyzed under dry nitrogen in standard heating mode from 25 °C to 800 °C at a heating rate of 20 °C/min. Changes in sample mass were recorded as a function of temperature. All measurements were performed in triplicate.

#### 2.14.3 Differential scanning calorimetry

Differential scanning calorimetry (DSC) was performed to assess the thermal transition profile of the hydrogel formulations. Dried hydrogel samples were placed in aluminum pans and analyzed using a DSC Diamond Series instrument (PerkinElmer, United States of America) under a nitrogen atmosphere. Samples were heated at a rate of 40 °C/min. The thermograms were used to identify endothermic and exothermic transitions associated with water loss, polymer relaxation and thermal degradation of the hydrogel network (Guinesi and Cavalheiro, 2006; Ravichandran et al., 2016).

### 2.15 Antibacterial activity

The antibacterial activity of Ch-HI, Ch/Rc-AgNP-HI and Ch/Ab-AgNP-HI was assessed against *E. coli* ATCC 25922 and *S. aureus* ATCC 25923 using the agar well diffusion method (Kumar et al., 2020; Talodthaisong et al., 2020; Wahid et al., 2020). Bacterial cultures were grown on blood agar (HBA) at 37 °C for 24 h. Single colonies were transferred into LB broth and incubated overnight. Overnight cultures were adjusted in LB broth to A600 nm = 0.10 ± 0.02, corresponding to approximately 1 × 10^8^ CFU/mL, and then diluted 1:100 in LB broth to obtain a working inoculum of approximately 1 × 10^6^ CFU/mL. Mueller-Hinton agar was prepared by pouring 25 mL autoclaved medium into sterile Petri plates and allowing the agar to solidify. Each plate was inoculated by spreading 100 µL of diluted bacterial suspension uniformly over the agar surface using a sterile spreader. Sterile Oxford cups were then placed into the agar.

Hydrogel samples were first incubated at 37 °C for 30 min to induce gelation before loading into the wells. Based on the formulation composition, the AgNP-loaded hydrogel sols contained 2.5 mg AgNPs in a final volume of 10 mL, corresponding to a nominal AgNP concentration of 250 μg/mL. Therefore, 50, 75 and 100 µL hydrogel aliquots contained approximately 12.5, 18.75 and 25 µg AgNPs, respectively. Blank Ch-HI was used as the formulation control and gentamicin was used as the positive antibacterial control. Plates were incubated at 37 °C for 24 h under aseptic conditions, zones of inhibition (mm) were measured. The assay was performed in triplicate.

### 2.16 *In vitro* cytocompatibility assay

The cytocompatibility of Rc-AgNPs, Ab-AgNPs, Ch-HI, Ch/Rc-AgNP-HI and Ch/Ab-AgNP-HI was assessed in spontaneously immortalized human foreskin fibroblasts (HFF-1) using an MTT cell viability assay. HFF-1 cells were maintained in Dulbecco’s modified Eagle medium (DMEM) supplemented with 10% fetal bovine serum and 1% penicillin-streptomycin at 37 °C in a humidified incubator with 5% CO_2_. Cells from passages 8-10 were used. Cells were seeded into 96-well plates at a density of 2 × 10^4^ cells/well and allowed to adhere for 24 h before treatment.

Test formulations were freshly prepared in DMEM and applied at AgNP-equivalent concentrations of 1–50 μg/mL. Blank Ch-HI was tested at equivalent hydrogel concentrations. Following 2 h exposure at 37 °C and 5% CO_2_, the treatment medium was removed, and cells were gently washed with PBS to eliminate residual formulation. Fresh medium containing MTT reagent was added, and the plates were incubated for a further 3 h in the dark to allow formazan formation. The MTT-containing medium was removed, formazan crystals were dissolved in 100 µL dimethyl sulfoxide (DMSO), and absorbance was measured at 570 nm using a microplate reader. Cell viability was calculated relative to untreated control cells. Untreated cells served as viability control, 0.1% (v/v) Triton X-100 was used as the cytotoxicity control, and the 70% viability threshold was used as a cytotoxicity reference according to ISO 10993-5 (Standardization, 2009).

### 2.17 *In vivo* burn wound healing evaluation

#### 2.17.1 Animal ethics and housing

All animal procedures were approved by the Institutional Animal Care and Use Committee of Shanxi Agricultural University, China (approval number SXAU-EAW-2023-M002003). Animal procedures and reporting were conducted in accordance with institutional ethical requirements and ARRIVE reporting principles where applicable. Female BALB/c wild-type mice (8–10 weeks old) were housed in the experimental animal laboratory of the College of Veterinary Medicine, Shanxi Agricultural University, China. Animals were maintained under controlled temperature, humidity and light conditions (23 °C ± 2 °C, 55% ± 5% relative humidity and 12 h light/dark cycle) with free access to standard diet, water and environmental enrichment. Mice were acclimatized before intervention, and all procedures involving animals and their care were performed according to institutional guidelines and relevant ethical requirements.

#### 2.17.2 Burn wound induction

After acclimatization, mice were randomly allocated into four groups (*n* = 10 animals/group; total *n* = 40): saline-treated burn control, Ch-HI, Ch/Rc-AgNP-HI and Ch/Ab-AgNP-HI. Animals were further allocated to predefined terminal sampling time points for histological and biochemical analyses. No serial wound biopsies were performed. Macroscopic wound images and clinical observations were collected longitudinally from animals while they remained in the study, up to their assigned endpoint.

For histological assessment, *n* = 4 animals per group were allocated across days 3, 7, 14 and 21, with one animal per group collected at each histological time point. For biochemical analyses, including hydroxyproline content and dry matter determination, *n* = 6 animals per group were allocated across days 7, 14 and 21, with two animals per group collected at each biochemical time point. Animals assigned to terminal tissue collection were euthanized at their designated sampling time points. The overall experimental workflow, including animal allocation, burn induction, treatment administration, monitoring and terminal tissue collection, is summarized in Figure 2.

**FIGURE 2:**
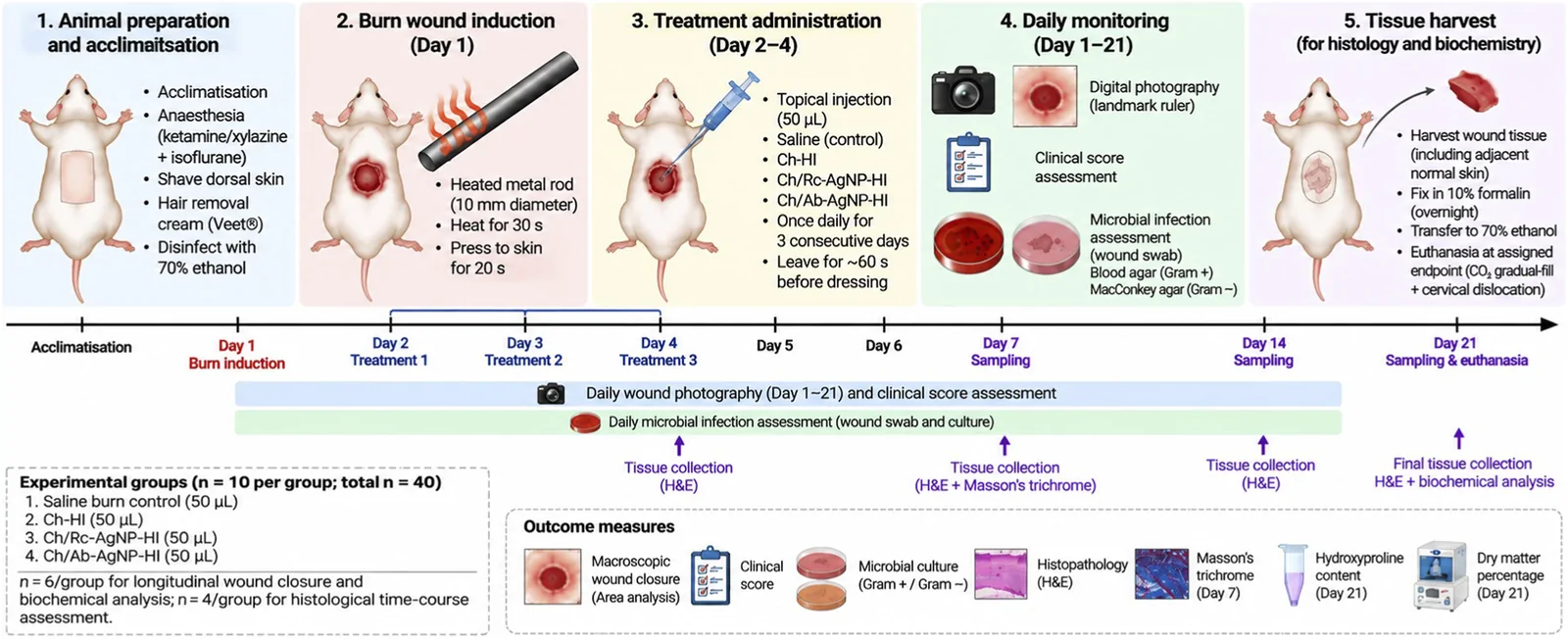
Experimental workflow for the *in vivo* burn wound healing study. Female BALB/c wild-type mice were acclimatized, second-degree burn wounds were induced on day 1, and animals received saline, Ch-HI, Ch/Rc-AgNP-HI or Ch/Ab-AgNP-HI once daily for three consecutive days. Wound progression and clinical scores were monitored from day 1 until the assigned endpoint, and microbial swabs were collected during the treatment period. The study used *n* = 10 animals per group. Of these, *n* = 4 animals per group were allocated for histological assessment across days 3, 7, 14 and 21, and *n* = 6 animals per group were allocated for hydroxyproline and dry matter analysis across days 7, 14 and 21. Animals assigned to terminal sampling were euthanized at their designated time points; no serial wound biopsies were performed. Tissue samples were collected at the assigned endpoints for H&E staining, Masson’s trichrome staining, hydroxyproline analysis and dry matter determination.

On the day of burn induction, mice were anesthetized by intraperitoneal administration of xylazine (10 mg/kg), ketamine (100 mg/kg) and buprenorphine (0.05 mg/kg, for analgesia) in 500 µL of 0.9% saline. Anesthesia was induced using 5% isoflurane in oxygen at 1.5 L/min and maintained with 2% isoflurane during the burn induction procedure. Mice were placed on a heat pad to maintain body temperature during the procedure.

Dorsal hair was removed using clippers and hair removal cream, and the shaved area was disinfected with 70% (v/v) ethanol. Second-degree burn wounds were induced according to a previously published protocol (Arafa et al., 2018) with slight modification. Briefly, placing a cylindrical metal rod with a 10 mm diameter on the dorsal skin surface for 20 s after heating the rod over an open flame for 30 s (Arafa et al., 2018). Clinical appearance of the wound and surrounding skin was monitored during the study period using a predefined clinical scoring system. The scoring criteria included wound appearance, erythema/edema, exudate, scab formation, animal behavior and signs of distress or abnormal wound progression. The clinical intervention threshold was defined before assessment, and animals meeting humane endpoint or intervention criteria were to receive appropriate intervention or be euthanized. No animals reached the predefined humane endpoint criteria during the study. The second-degree nature of the burn injury was confirmed histologically by H&E staining, showing dermal tissue disruption consistent with burn damage.

#### 2.17.3 Treatment administration

Animals received the assigned treatment directly on the wound bed: saline (50 µL), blank Ch-HI (50 µL), Ch/Rc-AgNP-HI (50 µL) or Ch/Ab-AgNP-HI (50 µL). For the AgNP-loaded hydrogel groups, 50 µL formulation delivered approximately 12.5 µg AgNPs per wound. Treatments were administered every 24 h for three consecutive days. After administration, the formulation was left undisturbed for approximately 60 s to support local retention before sterile adhesive dressings were reapplied. Sterile adhesive dressings, Opsite Flexigrid® (Smith & Nephew, UK), were used to cover the wound area and were replaced during treatment and monitoring as required. Wound healing progression, clinical appearance and animal wellbeing were monitored throughout the study period.

#### 2.17.4 Euthanasia and tissue collection

Animals meeting predefined humane endpoint criteria were to be euthanized immediately; however, no animals reached humane endpoint criteria during the study. Animals assigned to terminal tissue collection were euthanized at their predefined sampling time points on days 3, 7, 14 or 21, depending on the allocated endpoint. Euthanasia was performed by carbon dioxide (CO_2_) inhalation using a gradual-fill chamber method. CO_2_ was delivered at a displacement rate of 30% of the chamber volume per minute, equivalent to 0.30 chamber volumes/min, until respiration ceased. CO_2_ flow was continued for at least 1 min after respiratory arrest, and death was confirmed by cervical dislocation before tissue collection.

#### 2.17.5 Microbial infection assessment of induced burns

Surface swabs were collected from burn wounds before daily treatment applications to assess microbial contamination. The burned area was swabbed using a sterile cotton swab, focusing on the region with the greatest visible burn damage. Swab samples were homogenized in 4 mL sterile saline and immediately cultured on blood agar and MacConkey agar plates. MacConkey agar was used to assess Gram-negative bacterial growth, while blood agar was used for detection of Gram-positive bacteria. Plates were incubated at 37 °C for 24–48 h and inspected for bacterial growth.

#### 2.17.6 Macroscopic wound closure analysis

Digital photographs of wounds were taken throughout the study period on days 1, 3, 7, 14 and 21 for macroscopic assessment of wound healing. For animals assigned to earlier terminal tissue collection, wound images were included only up to their allocated endpoint. Animals assigned to the day 21 endpoint were followed throughout the full 21-day observation period. Images were calibrated against a measurement ruler and analyzed using ImageJ software (NIH, United States of America). Wound contraction was calculated relative to the initial wound area using Equation 8:

$$Wound\;contraction\;\left(\%\right)=\frac{Initial\;Wound\;area-Wound\;area\;on\;day\;n\;}{initial\;wound\;area\;}\times 100$$ (8)

where day n represents the wound area measured at each post-treatment time point.

#### 2.17.7 Histology and masson trichrome staining

For histological assessment, wound tissue containing epidermis, dermis, subcutaneous tissue and adjacent intact tissue was harvested at selected time points. Histological samples were collected from animals allocated to the histology cohort at days 3, 7, 14 and 21, with one animal per group collected at each time point. Samples were fixed in 10% neutral buffered formalin, dehydrated through graded ethanol, cleared with xylene, embedded in paraffin and sectioned at 4–5 µm thickness. Hematoxylin and eosin (H&E) staining was used to evaluate re-epithelialization, necrosis, inflammatory cell infiltration, granulation tissue formation and overall tissue organization. Masson trichrome staining was used to assess collagen deposition and extracellular matrix remodeling (Patil et al., 2018; Oryan et al., 2019).

#### 2.17.8 Hydroxyproline content and dry matter percentage

Dry matter percentage was determined by immediately weighing collected wound tissue samples, lyophilizing the samples and calculating dry weight relative to wet weight. Hydroxyproline content was measured using a Kiazist commercial kit according to the manufacturer’s instructions. Hydroxyproline was used as an indirect biochemical indicator of collagen-associated matrix deposition during wound repair (Patil et al., 2018; Oryan et al., 2019).

### 2.18 Statistical analysis

Data are presented as mean ± standard deviation (SD) unless otherwise stated. Experiments were performed in triplicate for *in vitro* and formulation studies unless otherwise indicated. Statistical analysis was performed using GraphPad Prism (version 10.6.1, 2025; GraphPad Software, Boston, MA, United States of America). For comparisons among multiple groups at a single time point, one-way analysis of variance (ANOVA) followed by Tukey’s *post hoc* test was used. For datasets involving two independent variables, such as treatment and temperature, treatment and concentration, or treatment and time, two-way ANOVA followed by an appropriate multiple-comparison test was used. A *p* value <0.05 was considered statistically significant. Figure captions define statistical notation used in the graphs: ns, not significant; *p < 0.05; **p < 0.01; ***p < 0.001; and ****p < 0.0001.

## 3 Results

### 3.1 Formulation pH and visual phase transition

Both AgNP-loaded hydrogel formulations showed pH values close to the physiological range before and after gelation (Table 1). The sol pH values were 7.12 ± 0.06 for Ch/Rc-AgNP-HI and 6.99 ± 0.05 for Ch/Ab-AgNP-HI, while the corresponding gel pH values were 7.35 ± 0.05 and 7.28 ± 0.04, respectively. The small increase in pH after gelation indicates that the formulation process and temperature-triggered transition did not produce a substantial pH shift. This is important for burn wound application because formulations with near-neutral pH are less likely to irritate exposed tissue than strongly acidic or alkaline preparations.

**TABLE 1:** Physicochemical characteristics of AgNP-loaded chitosan hydrogel injections (*n* = 3).

Formulation	pH (sol)	pH (gel)	Gelation temperature (°C)	Gelation time (s)	AgNP content (mg/10 mL)	EE (%)
Ch/Rc-AgNP-HI	7.12 ± 0.06	7.35 ± 0.05	37.2 ± 1.1	175 ± 1.4	2.37 ± 0.02	94.65 ± 0.6
Ch/Ab-AgNP-HI	6.99 ± 0.05	7.28 ± 0.04	36.8 ± 1.5	179 ± 1.4	2.28 ± 0.02	91.39 ± 0.7

AgNP, content was calculated from the initial AgNP, input of 2.5 mg per 10 mL formulation and the measured entrapment efficiency.

Visual evaluation demonstrated that Ch-HI, Ch/Rc-AgNP-HI and Ch/Ab-AgNP-HI were transparent to slightly opalescent at room temperature, indicating homogeneous dispersion of the hydrogel components in the sol state. After incubation at 37 °C, the formulations became turbid and formed non-flowing gels, confirming temperature-triggered network formation (Figure 3). This visible phase transition supports the intended application strategy in which the hydrogel can be administered as an injectable sol and then form a localized gel depot at body temperature.

**FIGURE 3:**
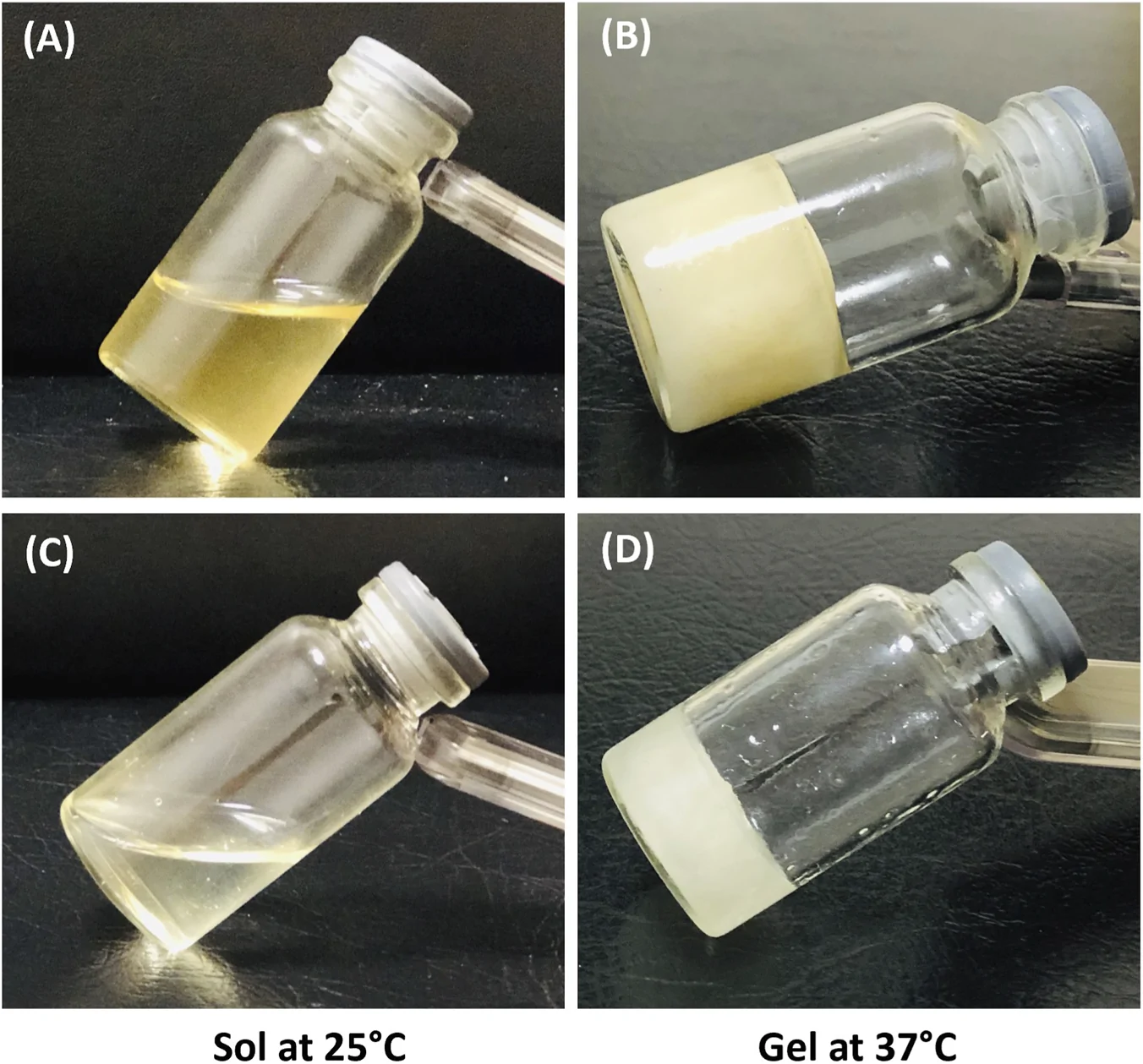
Visual phase transition of AgNP-loaded chitosan hydrogel injections at room temperature and 37 °C. The sol state at 25 °C and gel state at 37 °C demonstrate the thermosensitive behavior of the formulations. Images are representative of triplicate preparations (n = 3); no inferential statistical analysis was applied to photographic panels.

### 3.2 Optical transparency as a function of temperature

Optical transparency was measured to provide a quantitative readout of the temperature-responsive transition of Ch/Rc-AgNP-HI and Ch/Ab-AgNP-HI (Figure 4). At lower temperatures, both formulations showed high transmittance, consistent with homogeneous dispersion in the sol state. Ch/Rc-AgNP-HI retained approximately 100%, 97.8% and 95% transmittance at 10, 20 °C and 25 °C, respectively, while Ch/Ab-AgNP-HI showed approximately 100%, 96% and 88% transmittance at the same temperatures. These data indicate that both AgNP-loaded formulations remained optically transparent or slightly opalescent before thermally induced gelation.

**FIGURE 4:**
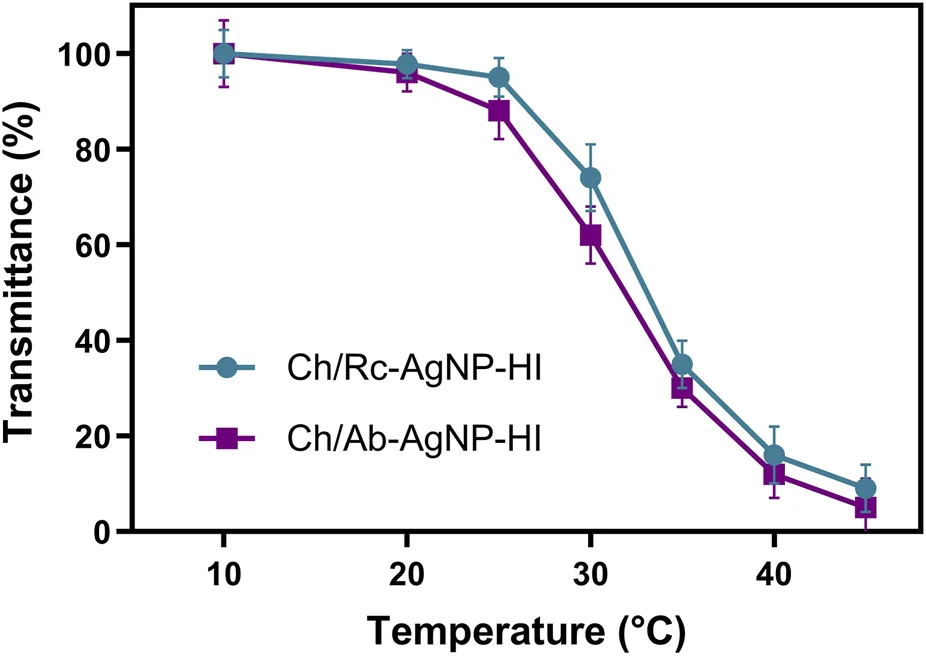
Optical transparency of Ch/Rc-AgNP-HI and Ch/Ab-AgNP-HI as a function of temperature. The reduction in transmittance with increasing temperature indicates thermosensitive sol-gel transition. Data are presented as mean ± SD (*n* = 3). Statistical comparisons between formulations across temperature were assessed by two-way ANOVA with multiple-comparison testing; where indicated, ns = not significant, *p < 0.05, **p < 0.01 and ***p < 0.001.

As temperature increased, transmittance progressively decreased, reflecting the transition from a clear injectable sol to a turbid hydrogel network. Ch/Rc-AgNP-HI showed transmittance values of approximately 74%, 35%, 16% and 9% at 30, 35, 40 °C and 45 °C, respectively. Ch/Ab-AgNP-HI showed a similar temperature-dependent reduction, with transmittance values of approximately 62%, 30%, 12% and 5% at the corresponding temperatures. The marked decline between 30 °C and 40 °C supports thermally induced network formation near the physiological temperature range, consistent with the visual gelation observations.

### 3.3 Sol-gel transition and physicochemical characteristics

The AgNP-loaded chitosan hydrogel formulations gelled rapidly at temperatures close to body temperature (Table 1). Ch/Rc-AgNP-HI and Ch/Ab-AgNP-HI exhibited gelation temperatures of 37.2 °C ± 1.1 °C and 36.8 °C ± 1.5 °C, respectively, confirming that incorporation of phytogenic AgNPs did not interfere with thermosensitive gel formation. Incipient gelation occurred within approximately 2–3 min, with gelation times of 175 ± 1.4 s for Ch/Rc-AgNP-HI and 179 ± 1.4 s for Ch/Ab-AgNP-HI. This rapid transition is advantageous for intrawound use because the formulation must remain fluid during administration but gel quickly enough to avoid loss from the wound bed.

Both formulations also showed efficient AgNP incorporation. Ch/Rc-AgNP-HI and Ch/Ab-AgNP-HI contained estimated retained AgNP amounts of 2.37 ± 0.02 and 2.28 ± 0.02 mg per 10 mL formulation, respectively, based on the initial AgNP input and measured entrapment efficiency. The corresponding EE values were 94.65% ± 0.6% and 91.39% ± 0.7%, respectively. These high EE values suggest that most of the added AgNPs were retained within the chitosan hydrogel network during preparation, supporting the suitability of this formulation approach for localized AgNP delivery.

### 3.4 Sol-gel fraction and texture properties

Sol-gel fraction analysis was performed to determine the relative stable gel network and soluble fraction of the prepared hydrogels (Figures 5A,B). All formulations showed high gel fractions and low sol fractions, indicating successful formation of hydrogel networks after thermally induced gelation. Although the AgNP-loaded formulations showed numerically comparable gel and sol fractions to blank Ch-HI, the differences were not statistically significant. These findings suggest that incorporation of Rc-AgNPs or Ab-AgNPs did not markedly disrupt chitosan/β-GP hydrogel network formation.

**FIGURE 5:**
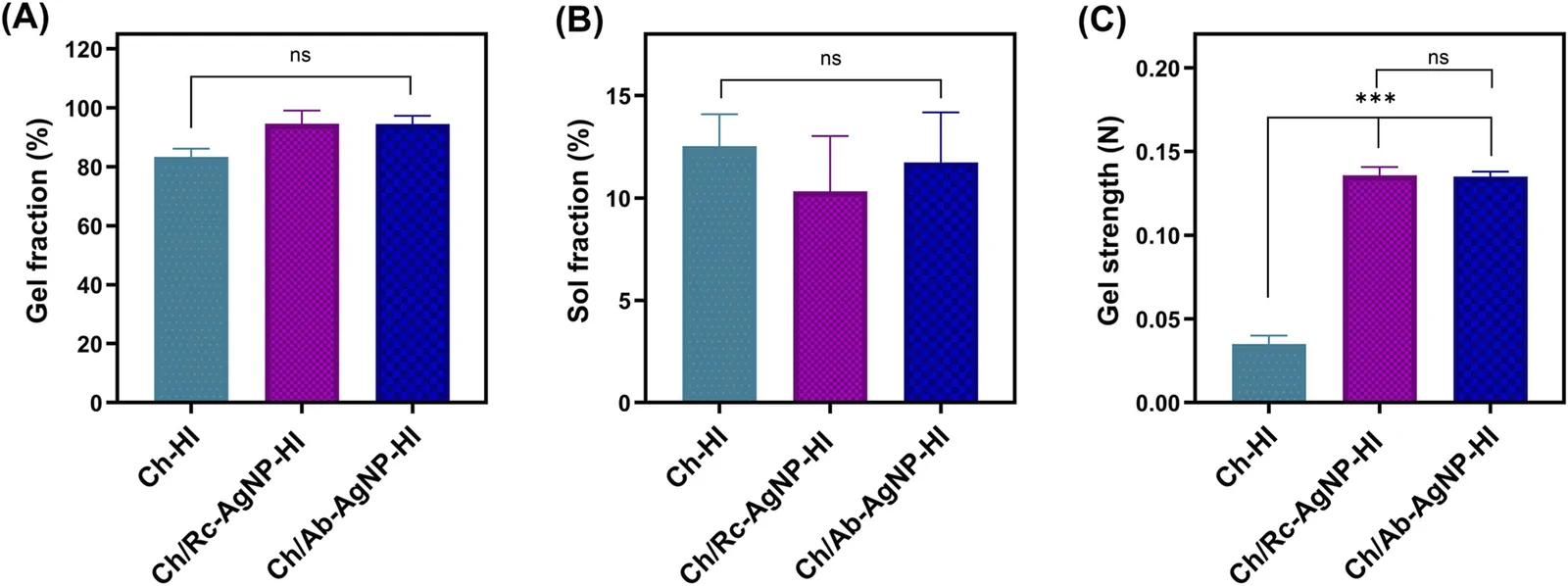
Sol-gel fraction and texture properties of Ch-HI, Ch/Rc-AgNP-HI and Ch/Ab-AgNP-HI. **(A)** Gel fraction, **(B)** sol fraction and **(C)** gel strength. Data are presented as mean ± SD (n = 3). Statistical significance was determined by one-way ANOVA with Tukey’s *post hoc* test; ns = not significant, *p* < 0.05, *p < 0.01 and **p < 0.001.

Texture analysis showed that AgNP incorporation increased the gel strength of the hydrogels (Figure 5C). Both Ch/Rc-AgNP-HI and Ch/Ab-AgNP-HI exhibited significantly higher gel strength than blank Ch-HI, indicating formation of mechanically more resistant hydrogel matrices after nanoparticle incorporation. No significant difference was observed between Ch/Rc-AgNP-HI and Ch/Ab-AgNP-HI, suggesting that both phytogenic AgNP systems produced comparable textural reinforcement under the tested conditions.

### 3.5 Rheological behavior

Rheological analysis was performed to evaluate the temperature-responsive viscoelastic behavior of the hydrogel formulations (Figure 6). During the temperature sweep, both AgNP-loaded hydrogels showed sol-like behavior at lower temperatures, followed by an increase in storage modulus (G′) after the transition region. The increase in G′ with temperature indicates progressive development of an elastic gel network as the formulations approached the physiological temperature range, supporting thermally induced sol–gel transition behavior consistent with previously reported thermoresponsive hydrogel systems (Maharjan et al., 2022). Transition behavior was observed at approximately 37.7 °C for Ch/Rc-AgNP-HI and 36.4 °C for Ch/Ab-AgNP-HI, in agreement with the vial inversion gelation results.

**FIGURE 6:**
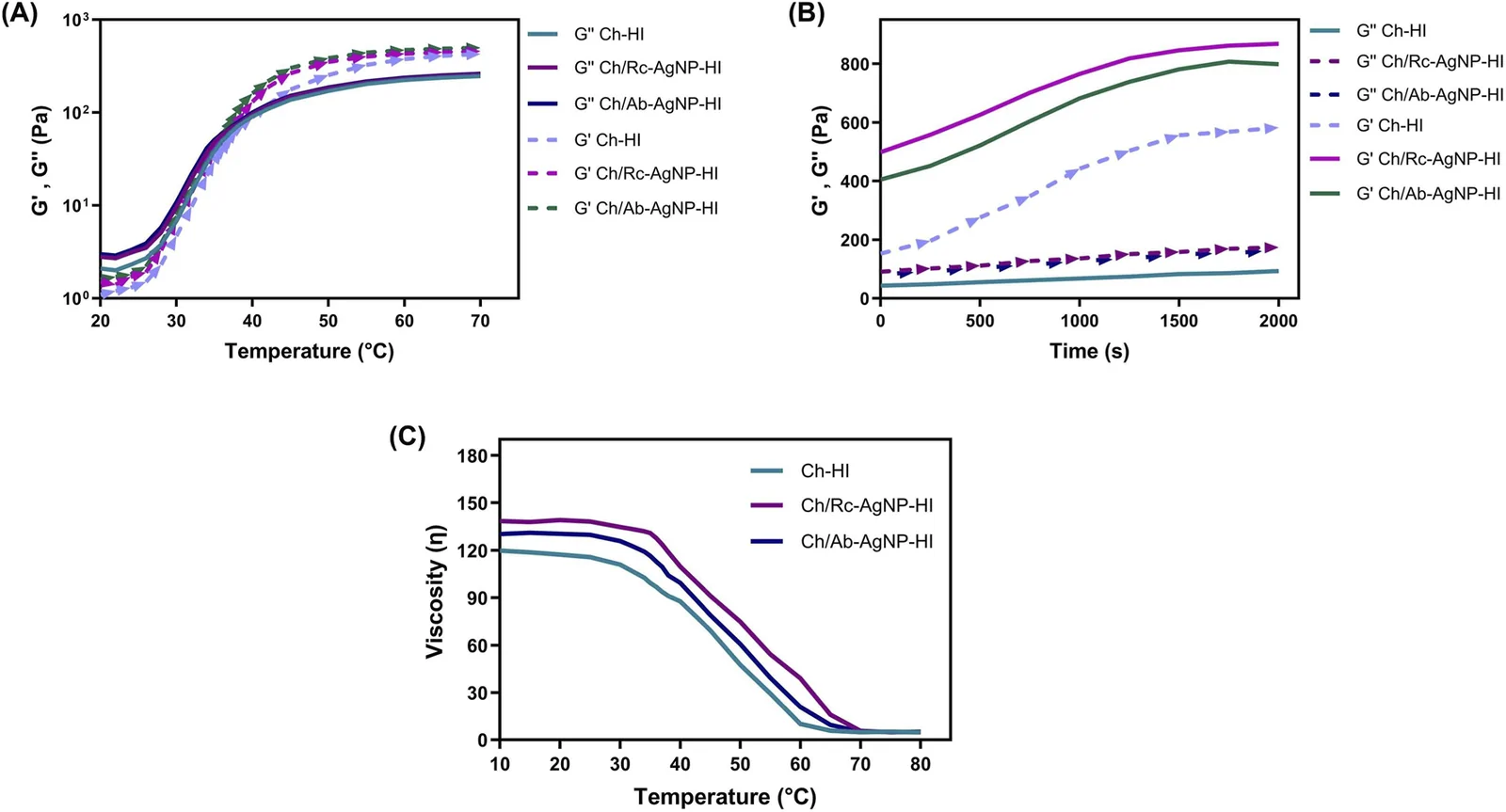
Rheological behavior of AgNP-loaded chitosan/β-GP hydrogel injections. **(A)** Storage modulus (G′) and loss modulus (G″) as a function of temperature, **(B)** G′ and G″ as a function of time at 37 °C and **(C)** viscosity as a function of temperature. Curves are representative of triplicate measurements or plotted as mean profiles where applicable (n = 3). Statistical comparisons were not applied to representative rheological profiles unless indicated.

At higher temperatures, G′ increased to approximately 461 Pa for Ch/Rc-AgNP-HI and 500 Pa for Ch/Ab-AgNP-HI, indicating strengthening of the gel network under the tested conditions. Viscosity decreased as temperature increased, suggesting improved flow behavior during heating before gel formation. However, quantitative injectability was not directly measured in this study and should be evaluated in future work using defined syringe and needle gauges. Time sweep analysis at 37 °C showed progressive network development over the measurement period, with G′ increasing from approximately 500–870 Pa for Ch/Rc-AgNP-HI and from approximately 400–800 Pa for Ch/Ab-AgNP-HI. Together, these rheological features support temperature-responsive network formation under the tested conditions, while further studies are required to quantify syringeability, extrusion force and long-term mechanical stability.

### 3.6 Swelling behavior

The AgNP-loaded hydrogels displayed temperature- and pH-responsive swelling behavior (Figure 7). At 25 °C, Ch/Rc-AgNP-HI and Ch/Ab-AgNP-HI showed swelling index values of approximately 9.5 and 9.3, respectively. When the temperature was increased to 37 °C, the swelling index decreased to approximately 7.5 for Ch/Rc-AgNP-HI and 7.0 for Ch/Ab-AgNP-HI. The differences between the two AgNP-loaded formulations were not significant at either temperature; however, the overall reduction in swelling at 37 °C was significant. This reduced swelling at physiological temperature may reflect thermally induced network formation and reduced polymer-chain relaxation after gelation.

**FIGURE 7:**
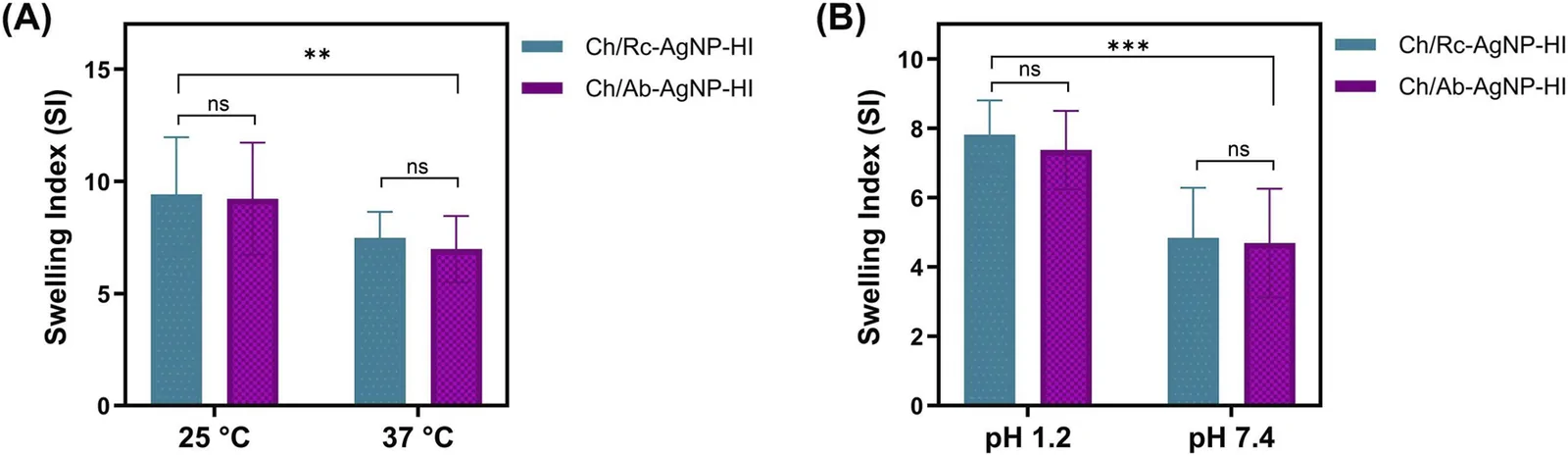
Swelling behavior of Ch/Rc-AgNP-HI and Ch/Ab-AgNP-HI. **(A)** Swelling index at 25 °C and 37 °C. **(B)** Swelling index at pH 1.2 and pH 7.4. Data are presented as mean ± SD (n = 3). Statistical significance was assessed by two-way ANOVA with multiple-comparison testing; ns = not significant, *p < 0.05 and **p < 0.01.

The hydrogels also showed pH-responsive swelling. Swelling was higher under acidic conditions at pH 1.2 than at pH 7.4. Ch/Rc-AgNP-HI and Ch/Ab-AgNP-HI showed swelling index values of approximately 7.8 and 7.4 at pH 1.2, respectively, compared with approximately 4.8 and 4.7 at pH 7.4. No significant difference was observed between the two formulations at the same pH, whereas the difference between acidic and neutral pH conditions was significant. This pH-dependent swelling is consistent with protonation of chitosan amine groups under acidic conditions, which can increase electrostatic repulsion and relaxation of the polymer network.

Overall, the swelling results indicate that both Ch/Rc-AgNP-HI and Ch/Ab-AgNP-HI retained responsive hydrogel behaviour. The reduced swelling at 37 °C supports formation of a more structured gel network at physiological temperature, while the enhanced swelling under acidic conditions suggests pH-sensitive hydration behaviour. For wound treatment, controlled swelling may assist wound-fluid interaction and diffusion of incorporated AgNPs while maintaining the hydrogel depot at the application site.

### 3.7 *In vitro* apparent Ag-containing species release and hydrogel erosion

The AgNP-loaded hydrogels showed pH-dependent apparent release of Ag-containing species over 30 h (Figures 8A,B). Under acidic conditions at pH 1.2, both Ch/Rc-AgNP-HI and Ch/Ab-AgNP-HI showed a progressive release profile, reaching approximately 90%–95% cumulative apparent release by 30 h. In contrast, release at pH 7.4 was slower and less extensive, with both formulations reaching approximately 55%–60% cumulative apparent release by 30 h. These findings indicate that acidic conditions promoted greater release of diffusible Ag-associated material from the hydrogel matrix than near-neutral conditions.

**FIGURE 8:**
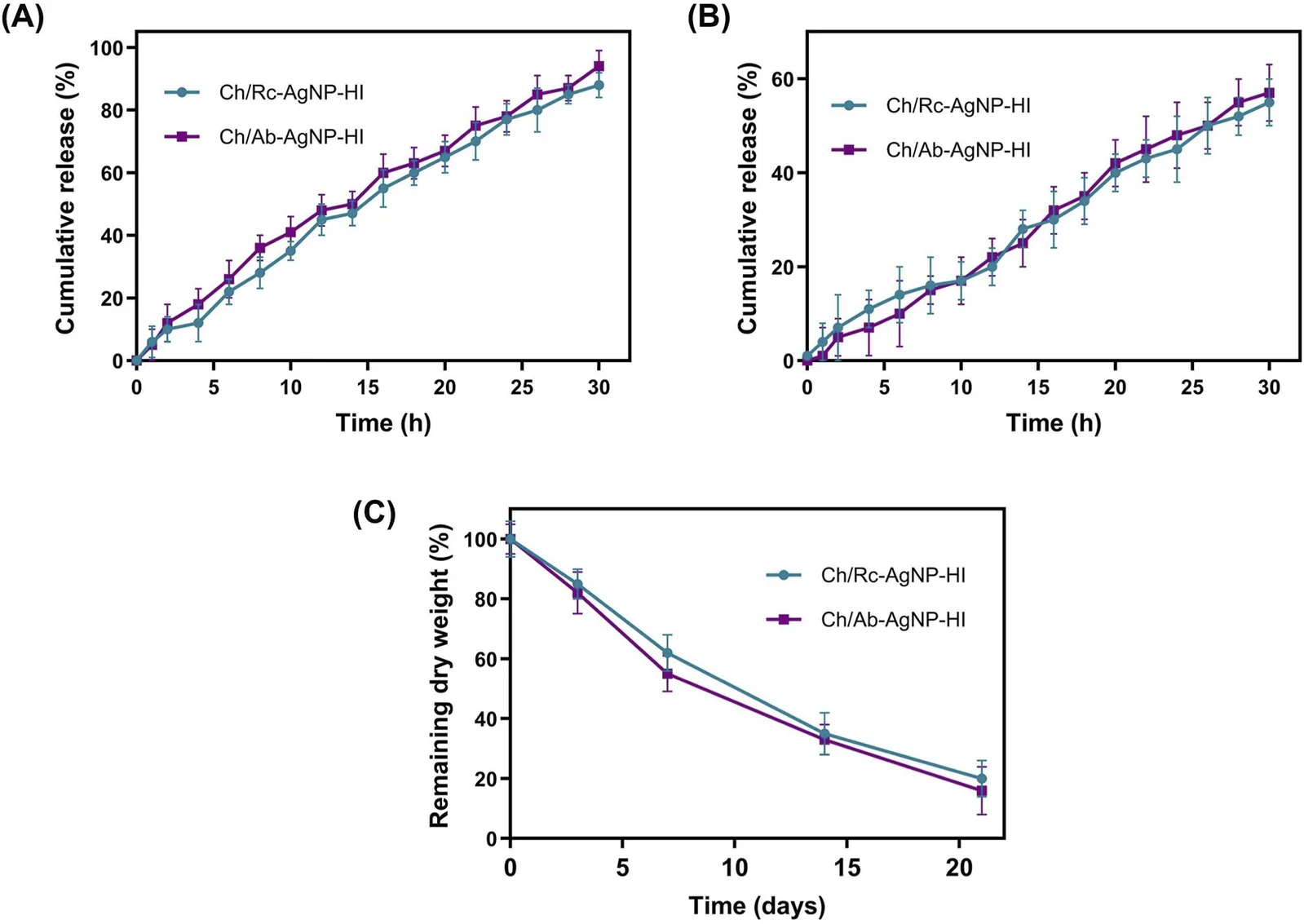
*In vitro* apparent Ag-containing species release and erosion behaviour of AgNP-loaded chitosan hydrogel injections. **(A)** Cumulative apparent release profile of diffusible Ag-containing species from Ch/Rc-AgNP-HI and Ch/Ab-AgNP-HI at pH 1.2 over 30 h. **(B)** Cumulative apparent release profile at pH 7.4 over 30 h. **(C)** *In vitro* erosion profile of Ch/Rc-AgNP-HI and Ch/Ab-AgNP-HI over 21 days. Release data should be interpreted as apparent release of diffusible Ag-containing species rather than confirmed release of intact AgNPs. Data are presented as mean ± SD (*n* = 3).

The release profiles showed an initial release phase followed by a slower release phase over the remaining study period, consistent with previously reported biphasic release behaviour from silver-based wound-healing systems (Singh et al., 2023). This pattern may reflect early diffusion of loosely associated or more readily diffusible Ag-containing species, followed by slower release governed by hydrogel hydration, matrix relaxation and diffusion through the chitosan/β-GP network. Because a 3.5 kDa dialysis membrane was used, the detected signal in the external release medium should not be interpreted as confirmed release of intact AgNPs. Rather, the data represent apparent release of diffusible Ag-containing species under the defined *in vitro* dialysis conditions.

The 30 h release window was selected to align with the early treatment period used in the *in vivo* study, where formulations were administered once daily for three consecutive days. The observed release behaviour suggests that the hydrogel matrix can provide continued availability of Ag-associated species over the first 24–30 h after application. However, these *in vitro* release conditions should be interpreted as comparative formulation data rather than a direct reproduction of the wound-bed microenvironment.

*In vitro* erosion studies showed gradual mass loss over 21 days (Figure 8C). The remaining hydrogel weight decreased from 100% at baseline to approximately 20% for Ch/Rc-AgNP-HI and 16% for Ch/Ab-AgNP-HI by day 21, indicating progressive matrix erosion after gel formation.

Together, the release and erosion data suggest that AgNP delivery from the injectable hydrogel system was governed by both diffusion through the swollen network and gradual hydrogel erosion. This release–erosion relationship is important for topical and intrawound delivery because it links formulation structure to local availability of AgNPs during the early treatment window and to subsequent matrix clearance during tissue repair.

### 3.8 Morphological and thermal characterization

SEM analysis was used to examine the freeze-dried morphology of Ch-HI and the AgNP-loaded hydrogels after lyophilization and fracture in liquid nitrogen (Figure 9A). The samples showed a porous-like dried architecture; however, this morphology should be interpreted as representative of the lyophilized hydrogel structure rather than the native hydrated gel network, because freeze-drying can introduce structural artefacts. Therefore, the SEM images support qualitative comparison of dried hydrogel morphology but do not directly confirm AgNP distribution within the hydrated gel matrix.

**FIGURE 9:**
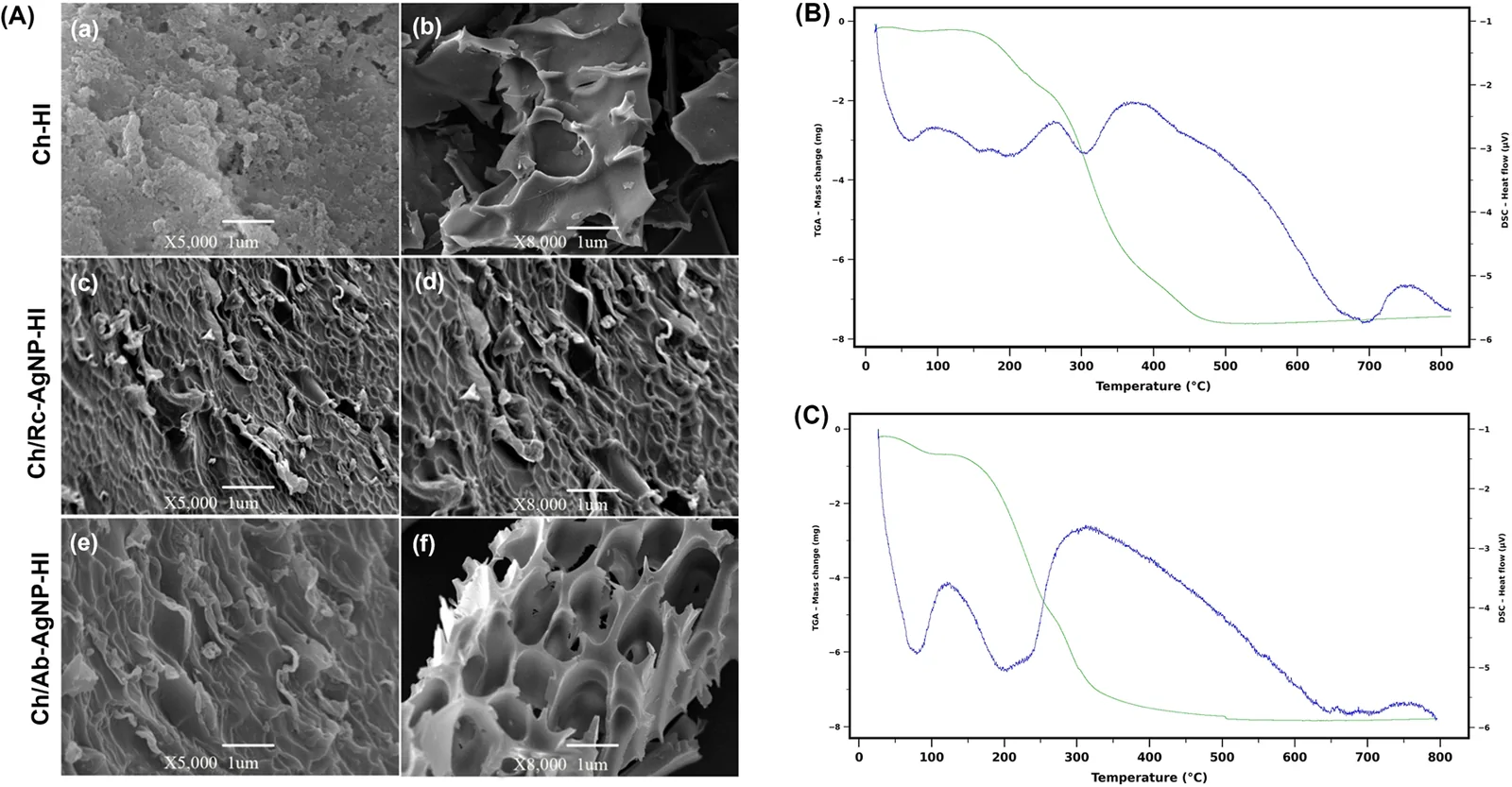
Morphological and thermal characterization of AgNP-loaded chitosan/β-GP hydrogels. **(A)** SEM micrographs of freeze-dried Ch-HI, Ch/Rc-AgNP-HI and Ch/Ab-AgNP-HI at matched magnifications. **(B)** TGA/DSC thermogram of Ch/Rc-AgNP-HI. **(C)** TGA/DSC thermogram of Ch/Ab-AgNP-HI. SEM images represent lyophilized hydrogel morphology and should not be interpreted as direct evidence of native hydrated gel porosity or AgNP distribution within the hydrated matrix. Representative SEM and thermal profiles are shown; no inferential statistical analysis was applied to these qualitative/representative panels.

Compared with blank Ch-HI, the AgNP-loaded formulations showed denser interconnected features in the freeze-dried state, which may reflect interactions between the incorporated AgNP-associated components and the chitosan/β-GP network. However, direct characterization of AgNP distribution within the hydrated hydrogel matrix by TEM, DLS/zeta potential, UV–Vis, XRD or related techniques was not performed in this study and should be addressed in future work.

Thermal analysis was used to compare the thermal behavior of the dried AgNP-loaded hydrogel formulations (Figures 9B,C). TGA profiles showed initial mass loss at lower temperatures, likely associated with physically adsorbed and bound water, followed by more pronounced mass reduction at elevated temperatures due to degradation of the chitosan-based polymer network and associated organic components. DSC thermograms showed broad thermal transitions accompanying dehydration and polymer degradation. No major thermal degradation event was observed near 37 °C, supporting compatibility with topical application at physiological temperature under the tested conditions. However, these thermal data should not be interpreted as evidence of long-term storage stability or sterilization compatibility, which were not directly assessed in this study.

### 3.9 Antibacterial activity

The AgNP-loaded hydrogel formulations demonstrated *in vitro* antibacterial activity against both *E. coli* and *S. aureus* in the agar diffusion assay. This assay supports the antibacterial potential of the formulations under controlled *in vitro* conditions. However, because the burn wound model did not show detectable bacterial growth in any group, these data should not be interpreted as evidence of *in vivo* antibacterial efficacy. Ch/Rc-AgNP-HI and Ch/Ab-AgNP-HI exhibited antibacterial activity against both *E. coli* ATCC 25922 and *S. aureus* ATCC 25923 (Table 2; Figure 10). Inhibition zones increased with increasing applied hydrogel volume, corresponding to increasing nominal AgNP dose. At 50, 75 and 100 μL, the AgNP-loaded hydrogel aliquots contained approximately 12.5, 18.75 and 25 µg AgNPs, respectively. This dose-dependent trend indicates that AgNPs retained antibacterial functionality after incorporation into the hydrogel network.

**TABLE 2:** Zone of inhibition (mm) of AgNP-loaded hydrogel injections, blank hydrogel and gentamicin against pathogenic bacterial strains (*n* = 3).

Bacterial strain	Ch/Rc-AgNP-HI			Ch/Ab-AgNP-HI			Ch-HI	Gentamicin
	50 μL	75 μL	100 μL	50 μL	75 μL	100 μL		
*E. coli* ATCC 25922	15.6 ± 0.43	17.6 ± 0.93	20.4 ± 0.65	15.35 ± 0.85	16.4 ± 0.85	19.3 ± 0.35	9.3 ± 0.46	25.54 ± 0.35
*S. aureus* ATCC 25923	16.56 ± 0.26	18.8 ± 0.75	22.5 ± 0.36	16.23 ± 0.46	18.4 ± 0.64	21.3 ± 0.47	10.6 ± 0.74	26.35 ± 0.38

**FIGURE 10:**
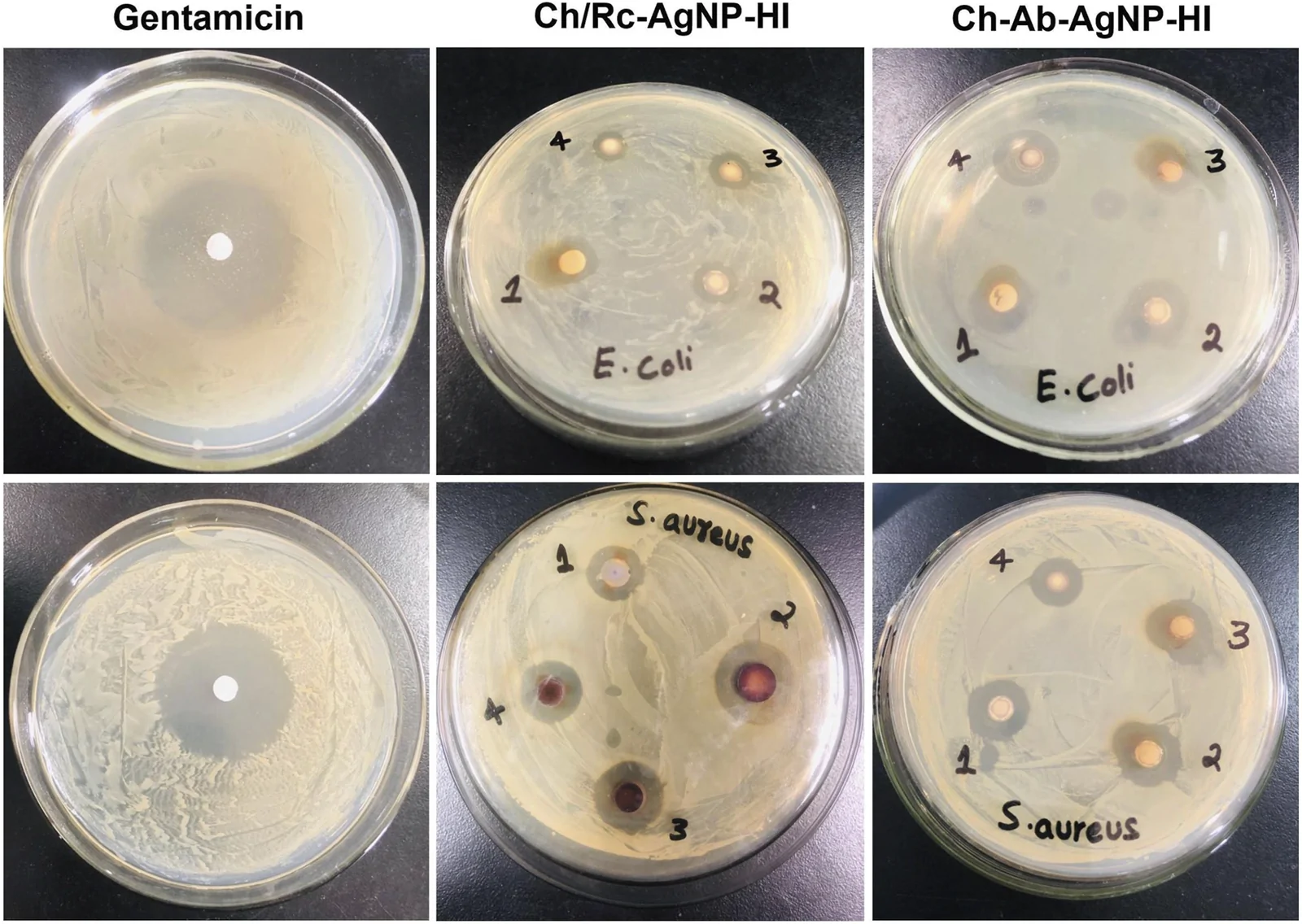
*In vitro* antibacterial activity of Ch/Rc-AgNP-HI and Ch/Ab-AgNP-HI against *E. coli* ATCC 25922 and *S. aureus* ATCC 25923 using the agar well diffusion assay. Hydrogel volumes were 50 µL (1), 75 µL (2) and 100 µL (3), corresponding to approximately 12.5, 18.75 and 25 µg AgNPs, respectively. Blank Ch-HI served as the formulation control (4), and gentamicin served as the positive control. Representative agar plates are shown; quantitative zone-of-inhibition values are reported in Table 2.

Against *E. coli* ATCC 25922, Ch/Rc-AgNP-HI produced inhibition zones of 15.6 ± 0.43, 17.6 ± 0.93 and 20.4 ± 0.65 mm at 50, 75 and 100 μL, respectively, whereas Ch/Ab-AgNP-HI produced zones of 15.35 ± 0.85, 16.4 ± 0.85 and 19.3 ± 0.35 mm at the same volumes. Against *S. aureus* ATCC 25923, Ch/Rc-AgNP-HI produced inhibition zones of 16.56 ± 0.26, 18.8 ± 0.75 and 22.5 ± 0.36 mm, while Ch/Ab-AgNP-HI produced 16.23 ± 0.46, 18.4 ± 0.64 and 21.3 ± 0.47 mm, respectively. Gentamicin produced the largest zones, as expected for the positive control, while blank Ch-HI showed lower activity than AgNP-loaded hydrogels. The slightly larger inhibition zones produced by Ch/Rc-AgNP-HI suggest stronger antibacterial diffusion or activity under the tested conditions.

The visible diffusion zones around AgNP-loaded hydrogel wells further support release of antibacterial components from the gel matrix into the agar. Overall, these results indicate that the hydrogel platform preserved the antibacterial activity of the phytogenic AgNPs and provided volume-dependent inhibition of representative Gram-negative and Gram-positive bacteria.

### 3.10 Cytocompatibility in HFF-1 fibroblasts

The short-term cytocompatibility of Rc-AgNPs, Ab-AgNPs, Ch-HI, Ch/Rc-AgNP-HI and Ch/Ab-AgNP-HI was evaluated in HFF-1 fibroblasts using an MTT assay after 2 h exposure over an AgNP-equivalent concentration range of 1–50 μg/mL (Figure 11). Across the tested concentrations, cell viability remained above the 70% threshold commonly used for preliminary *in vitro* cytotoxicity assessment. Most treatment groups maintained viability close to or above 90% relative to untreated controls under these short-term conditions. At the highest concentration of 50 μg/mL, a small reduction in viability was observed in some groups; however, viability remained above the cytotoxicity threshold.

**FIGURE 11:**
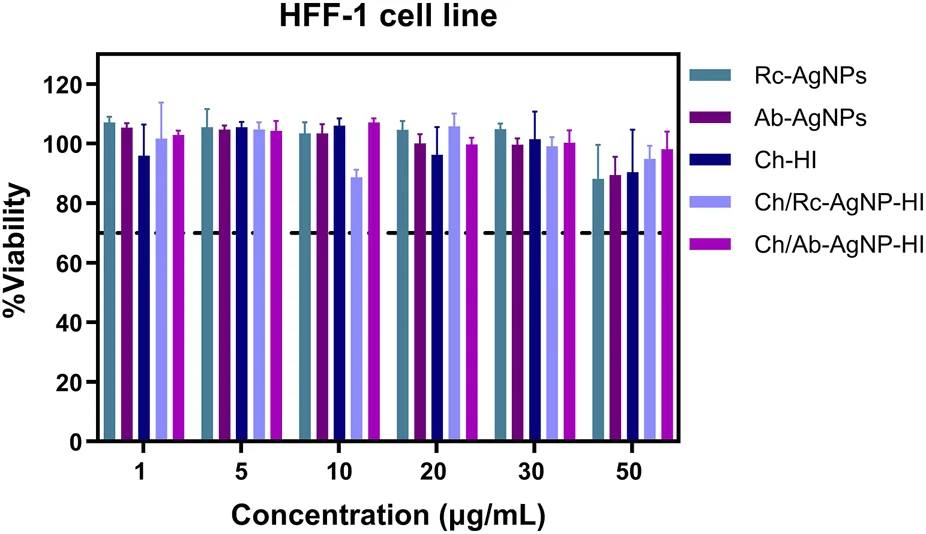
Cytocompatibility of Rc-AgNPs, Ab-AgNPs, Ch-HI, Ch/Rc-AgNP-HI and Ch/Ab-AgNP-HI in human foreskin fibroblast (HFF-1) cells. Cells were exposed to 1–50 μg/mL formulations, and viability was assessed by MTT assay. The dashed line indicates the 70% cell viability threshold. Data are presented as mean ± SD (*n* = 3).

These findings suggest that the AgNP-loaded hydrogels did not markedly reduce HFF-1 metabolic activity during the 2 h exposure period. However, the data should be interpreted as a preliminary short-term fibroblast compatibility screen rather than definitive evidence of long-term cytocompatibility or safety. Silver-associated cytotoxicity can depend on exposure duration, AgNP concentration, silver ion release, cell type and assay format. Therefore, longer exposure periods, additional skin-relevant cell types such as keratinocytes, and complementary assays such as Live/Dead staining and LDH release are required to more comprehensively evaluate cellular safety.

### 3.11 Microbial surveillance of induced burn wounds

Microbial swab assessment showed no detectable bacterial growth on blood agar or MacConkey agar plates from any treatment group during the observation period. Therefore, the burn wound model used in this study was considered a non-infected burn wound repair model with microbial surveillance. These findings indicate that the *in vivo* study was not designed to evaluate therapeutic antibacterial efficacy against an established wound infection. Accordingly, antibacterial activity of the AgNP-loaded hydrogels was interpreted based on the *in vitro* agar diffusion assay, while the animal study was interpreted in terms of wound closure, tissue repair and local tolerability.

### 3.12 Macroscopic burn wound healing and clinical monitoring

Macroscopic wound evaluation showed progressive healing in all groups, but wound closure was accelerated in the AgNP-loaded hydrogel-treated groups (Figures 12A,B). At day 3, wound contraction was 6.67% in the saline-treated burn control group, 9.43% in the Ch-HI group, 17.34% in the Ch/Rc-AgNP-HI group and 16.67% in the Ch/Ab-AgNP-HI group. This early difference suggests that AgNP-loaded hydrogel treatment began to influence wound closure within the first week after burn injury.

**FIGURE 12:**
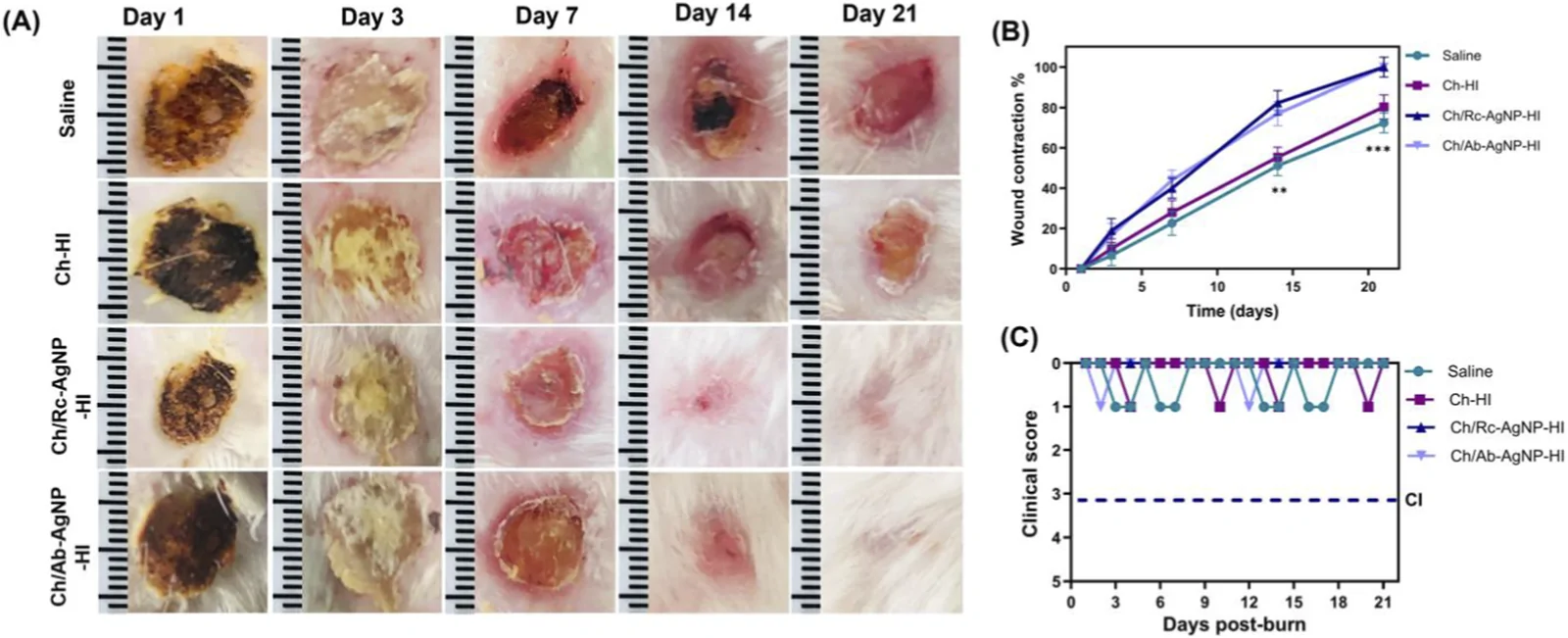
Macroscopic burn wound healing and clinical monitoring after treatment with saline, Ch-HI, Ch/Rc-AgNP-HI and Ch/Ab-AgNP-HI in female BALB/c wild-type mice. **(A)** Representative wound photographs taken on days 1, 3, 7, 14 and 21 post-burn. **(B)** Quantitative wound contraction analysis over the study period. **(C)** Clinical score monitoring used to assess animal wellbeing and treatment tolerability. The dashed line indicates the predefined clinical intervention threshold described in the Methods. Wound contraction data are presented as mean ± SD from animals remaining in the study at each time point according to the predefined terminal sampling schedule. Statistical significance for wound contraction was assessed by two-way ANOVA with multiple-comparison testing; ns = not significant, *p* < 0.05, *p < 0.01 and **p < 0.001. Clinical scores were monitored descriptively throughout the study period.

By day 7, the AgNP-loaded hydrogel groups showed markedly greater wound closure, reaching 57.54% and 55.20% for Ch/Rc-AgNP-HI and Ch/Ab-AgNP-HI, respectively, compared with 26.67% in the saline control and 24.54% in the Ch-HI group. By day 14, Ch/Rc-AgNP-HI and Ch/Ab-AgNP-HI achieved near-complete wound contraction of 99.34% ± 0.4% and 98.75% ± 0.6%, respectively, whereas the saline control and Ch-HI groups showed slower closure. Complete macroscopic closure was observed in both AgNP-loaded hydrogel groups by day 21. These findings indicate that AgNP-loaded chitosan hydrogel injections promoted faster macroscopic wound closure under the conditions of this non-infected burn model. However, wound contraction represents only one component of wound repair and should be interpreted alongside the histological and biochemical findings.

Clinical scoring was included to assess animal wellbeing and treatment tolerability during the *in vivo* study (Figure 12C). Across all groups, clinical scores remained low throughout the 21-day monitoring period and did not approach the predefined clinical intervention threshold. No treatment-related increase in clinical score was observed in mice treated with Ch/Rc-AgNP-HI or Ch/Ab-AgNP-HI compared with saline or blank Ch-HI. This indicates that the accelerated wound closure observed with the AgNP-loaded hydrogels was not associated with observable systemic distress or adverse clinical signs under the dosing conditions used in this study.

### 3.13 Histological evaluation by H&E and masson trichrome staining

H&E staining was used to assess re-epithelialization, inflammatory cell infiltration, necrotic features, granulation tissue development and overall tissue organization during burn wound healing (Figure 13A). Saline-treated burn control wounds showed persistent tissue disruption, inflammatory infiltration and delayed epidermal restoration across the observation period. Ch-HI treatment partially improved tissue organization compared with saline control, consistent with the wound-supportive properties of the chitosan matrix.

**FIGURE 13:**
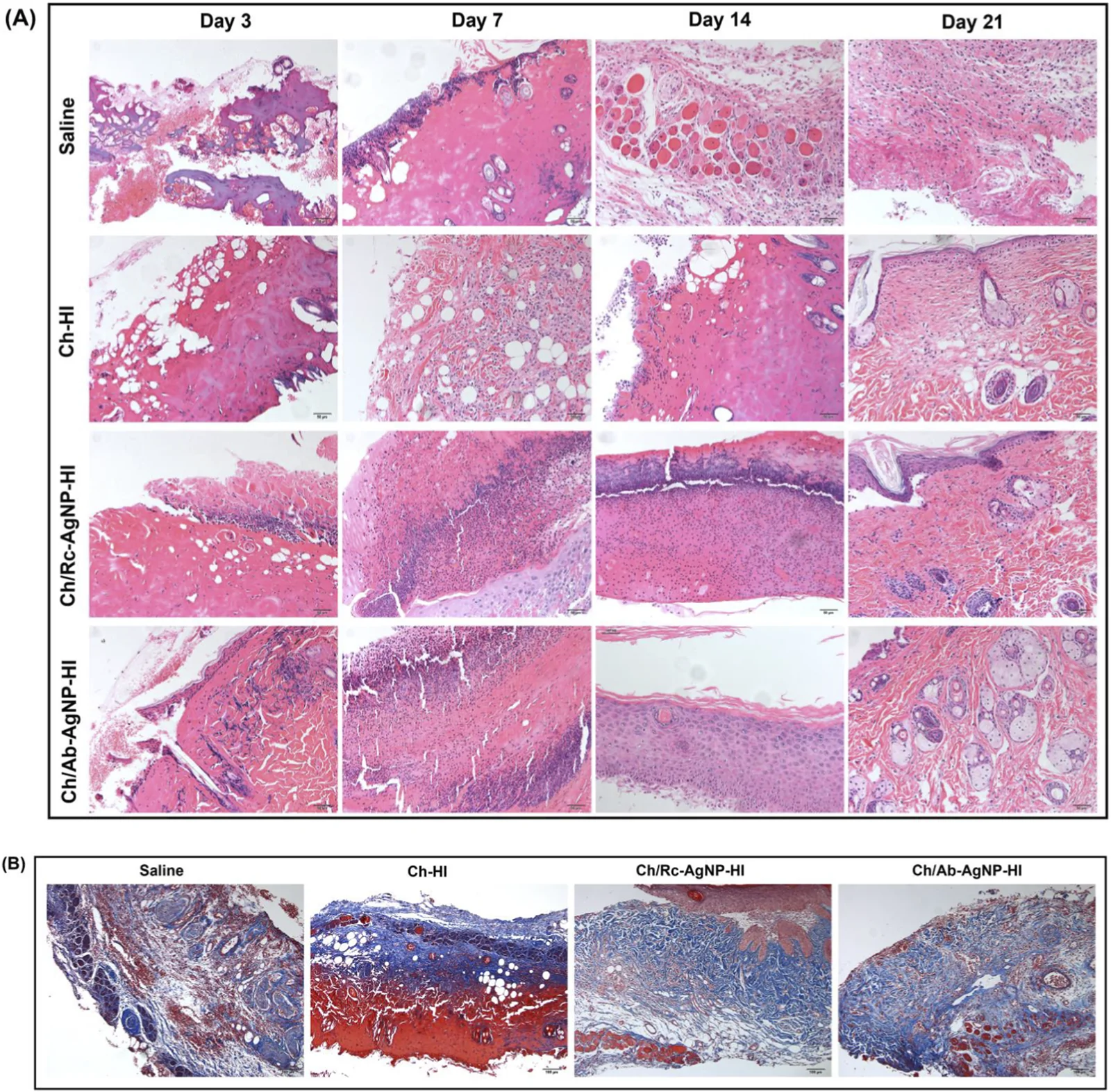
Histological and collagen-staining assessment of burn wound repair. **(A)** H&E staining of wound tissue after treatment with saline, Ch-HI, Ch/Rc-AgNP-HI and Ch/Ab-AgNP-HI; columns represent days 3, 7, 14 and 21, and rows represent treatment groups. **(B)** Masson trichrome staining of wound tissue showing collagen deposition and extracellular matrix organization in the treatment groups. Images are representative of the indicated samples; no inferential statistical analysis was applied to qualitative histology panels unless quantitative histomorphometry is added.

In contrast, Ch/Rc-AgNP-HI- and Ch/Ab-AgNP-HI-treated wounds showed more advanced tissue repair, particularly by days 14 and 21. The AgNP-loaded hydrogel groups exhibited improved epidermal organization, reduced inflammatory appearance and more organized dermal architecture compared with saline control and blank Ch-HI. These histological observations were consistent with the accelerated wound contraction observed macroscopically.

Masson trichrome staining was used as a complementary histological assessment of collagen deposition and extracellular matrix organization (Figure 13B). Compared with saline control and blank Ch-HI, wounds treated with AgNP-loaded hydrogels showed more organized collagen staining, supporting enhanced matrix restoration during the repair process. The improved collagen organization observed in the Ch/Rc-AgNP-HI and Ch/Ab-AgNP-HI groups aligned with the wound closure, H&E and biochemical findings, suggesting that these formulations supported both surface closure and collagen-associated dermal remodeling.

### 3.14 Hydroxyproline content and dry matter percentage

Hydroxyproline content and dry matter were used as supportive biochemical indicators of collagen deposition and tissue recovery (Table 3). The data should be interpreted together with macroscopic wound closure and histological findings, rather than as standalone mechanistic evidence of regeneration. Hydroxyproline content increased progressively in the saline-treated burn control group from 14.20 ± 0.45 μg/mg skin on day 7–18.65 ± 0.52 μg/mg skin on day 14 and 22.45 ± 0.53 μg/mg skin on day 21, indicating gradual endogenous repair. Ch-HI improved hydroxyproline content compared with saline control, reaching 16.50 ± 0.38, 23.35 ± 0.23 and 28.53 ± 0.24 μg/mg skin on days 7, 14 and 21, respectively.

**TABLE 3:** Hydroxyproline content and dry matter analysis of burn wound tissues.

Treatment	Total hydroxyproline (μg/mg skin)			Dry matter (%)		
​	Day 7	Day 14	Day 21	Day 7	Day 14	Day 21
Saline	14.20 ± 0.45	18.65 ± 0.52	22.45 ± 0.53	22.50 ± 0.63	24.76 ± 0.45	26.45 ± 0.85
Ch-HI	16.50 ± 0.38	23.35 ± 0.23	28.53 ± 0.24	23.95 ± 0.26	25.64 ± 0.25	28.76 ± 0.81
Ch/Rc-AgNP-HI	20.30 ± 0.54	31.34 ± 0.45	34.82 ± 0.48	29.56 ± 0.73	33.76 ± 0.64	35.42 ± 0.58
Ch/Ab-AgNP-HI	18.75 ± 0.42	27.48 ± 0.76	34.15 ± 0.51	25.84 ± 0.76	30.25 ± 0.55	33.68 ± 0.62

Hydroxyproline content and dry matter were determined from animals allocated to biochemical tissue collection on days 7, 14 and 21, with two animals per group per time point. Data are presented as mean ± SD., Statistical comparisons were performed using one-way ANOVA, with *p* < 0.05 considered statistically significant.

The AgNP-loaded hydrogels produced a more pronounced biochemical repair response. Ch/Rc-AgNP-HI showed the highest hydroxyproline content on days 7 and 14, reaching 20.30 ± 0.54 and 31.34 ± 0.45 μg/mg skin, respectively, consistent with its rapid early-to-mid wound contraction. By day 21, hydroxyproline levels remained high in both AgNP-loaded groups, reaching 34.82 ± 0.48 μg/mg skin for Ch/Rc-AgNP-HI and 34.15 ± 0.51 μg/mg skin for Ch/Ab-AgNP-HI. These results indicate enhanced collagen-associated repair in the AgNP-loaded hydrogel groups relative to saline control and blank hydrogel.

A similar trend was observed for dry matter percentage. Ch/Rc-AgNP-HI showed the highest dry matter values across the evaluated time points, reaching 29.56% ± 0.73%, 33.76% ± 0.64% and 35.42% ± 0.58% on days 7, 14 and 21, respectively, compared with 22.50% ± 0.63%, 24.76% ± 0.45% and 26.45% ± 0.85% in the saline control group. Ch/Ab-AgNP-HI also improved dry matter recovery, reaching 25.84% ± 0.76%, 30.25% ± 0.55% and 33.68% ± 0.62% on days 7, 14 and 21, respectively. These biochemical data strengthen the wound closure and histological findings by demonstrating enhanced collagen-associated matrix deposition and tissue regeneration in AgNP-loaded hydrogel-treated wounds.

## 4 Discussion

This study demonstrates that previously characterized phytogenic AgNPs can be incorporated into injectable chitosan/β-GP thermosensitive hydrogels and evaluated as local burn wound repair platforms with *in vitro* antibacterial potential. The formulation-to-function design is clinically relevant because burn wounds require materials that can conform to irregular tissue surfaces, limit microbial exposure, maintain hydration and support repair without repeated traumatic manipulation (Cook et al., 2022).

The injectable thermosensitive format directly addresses several of these needs. Injectable chitosan-based hydrogels can be administered as low-viscosity sols and then form local depots after exposure to physiological temperature, enabling intimate contact with wound contours and reducing formulation loss from the wound bed (Li et al., 2024; Wang et al., 2025). Recent reviews of chitosan hydrogel dressings further emphasize that chitosan contributes biodegradability, mucoadhesion, hemostatic activity and intrinsic antimicrobial effects, which are desirable for skin wound treatment (Guo et al., 2024; Li et al., 2024).

The choice of *R. communis* and *A. barbadensis* as plant sources for AgNP synthesis also supports the biological rationale of the platform. Plant-mediated AgNP synthesis uses phytochemicals as reducing and stabilizing agents, which can influence particle surface chemistry, colloidal behavior and biological activity (Dhir et al., 2024; Gou et al., 2024). In our previous study, Rc-AgNPs and Ab-AgNPs were successfully synthesized and shown to possess physicochemical and biomedical properties relevant to antimicrobial applications (Ahsan et al., 2025). Aloe-based materials are additionally well represented in wound and burn literature, with hydrogel-focused and clinical burn reviews describing their relevance to inflammation, tissue repair and burn management (Cook et al., 2022; Aldakheel et al., 2023; Imchen et al., 2025).

The pH, visual transition and optical transparency results confirmed that the formulations were homogeneous in the sol state and became turbid after temperature-triggered gelation. The sharp fall in transmittance as the temperature approached the physiological range is consistent with formation of a denser hydrogel network and supports the intended use of the formulation as an injectable in situ-forming depot (Khan et al., 2018; Xue et al., 2020). Near-neutral pH before and after gelation is also important for wound application because extreme acidity or alkalinity can irritate exposed tissue and interfere with the wound-healing microenvironment (Cook et al., 2022; Li et al., 2024).

Gelation occurred within approximately 2–3 min at temperatures close to body temperature, suggesting that AgNP incorporation did not compromise the thermosensitive behavior of the chitosan system. Rapid gelation is desirable for intrawound use because the formulation must be fluid during administration but should gel quickly enough to remain localized after application (Li et al., 2024). The high AgNP entrapment efficiencies further indicate that the chitosan/glycerol matrix was able to retain most of the added AgNPs during formulation, supporting its suitability as a local AgNP delivery vehicle (Haidari et al., 2021b; He et al., 2025).

Sol-gel fraction, texture and rheological data provide complementary evidence of hydrogel network formation. The increased gel fraction and reduced sol fraction in the AgNP-loaded hydrogels indicate formation of a more stable network with fewer soluble components. The texture data showed that AgNP incorporation significantly modified the mechanical response of the gels, while rheological analysis confirmed temperature-responsive conversion from sol-like to more elastic gel-like behavior (Zaragoza et al., 2018; Alinejad et al., 2019; Kocak et al., 2020). Nanoparticles can alter hydrogel mechanics through particle-polymer interactions, changes in network density and modification of water-polymer interactions, so mechanical effects should be interpreted in relation to the complete formulation rather than as a simple additive effect (Zaragoza et al., 2018; Guo et al., 2024).

The swelling data further support formation of a responsive hydrogel network. Both AgNP-loaded formulations showed lower swelling at 37 °C than at 25 °C, which is consistent with temperature-induced gel network formation and reduced chain mobility after gelation. This behaviour may be advantageous for intrawound application because excessive swelling at body temperature could weaken local retention or distort the wound interface, whereas a more structured gel can remain localized after administration. The higher swelling observed at pH 1.2 compared with pH 7.4 is consistent with protonation of chitosan amine groups, increased electrostatic repulsion and enhanced hydration of the polymer network under acidic conditions (Rehmani et al., 2017; Li et al., 2018). For wound treatment, controlled swelling may support fluid interaction, hydrogel hydration and diffusion of incorporated active components without compromising depot integrity (Kocak et al., 2020; Guo et al., 2024).

The release study showed biphasic apparent release of Ag-containing species over 30 h at pH 1.2 and pH 7.4, with an initial release phase followed by a slower sustained phase. The acidic and near-neutral media were used to probe pH-responsive release behaviour and should be understood as *in vitro* release conditions rather than direct reproduction of the wound-bed environment. The early phase may reflect the release of more readily diffusible Ag-associated species or weakly retained material near the hydrogel surface, whereas the slower phase is consistent with diffusion through the hydrated chitosan/β-GP matrix and progressive network relaxation (Rehmani et al., 2017; Haidari et al., 2021b).

The 30 h release window is relevant to the dosing schedule used in the mouse burn model, where formulations were administered every 24 h for three consecutive days. A formulation that provides continued availability of Ag-associated species across this time window may help maintain local exposure between applications while reducing reliance on a purely immediate burst effect (Li et al., 2018; He et al., 2025). The accompanying erosion data showed progressive hydrogel mass loss over 21 days, indicating that matrix erosion may also contribute to the gradual liberation of incorporated Ag-associated material and subsequent clearance of the hydrogel from the tissue site (Chee et al., 2018). However, because the dialysis membrane had a 3.5 kDa molecular weight cut-off and UV–visible spectroscopy was used for detection, these data should be interpreted as apparent release of diffusible Ag-containing species rather than confirmed release of intact AgNPs. Future studies using ICP-MS or AAS would be required to quantify total silver release and distinguish between ionic silver, nanoparticulate silver and other Ag-associated species.

SEM analysis supported formation of porous hydrogel architectures after freeze-drying and fracture. Porosity is useful for wound-facing materials because it can support water uptake, fluid exchange and transport of active components, although freeze-dried SEM morphology should be interpreted as a structural representation rather than a direct image of the fully hydrated gel state (Cook et al., 2022; Guo et al., 2024). Thermal analysis showed no major degradation event near 37 °C, supporting the suitability of the formulations for physiological-temperature application (Guinesi and Cavalheiro, 2006; Javaid et al., 2018).

The agar well diffusion results showed that incorporation into the chitosan hydrogel matrix preserved the *in vitro* antibacterial activity of Rc-AgNPs and Ab-AgNPs against both *E. coli* ATCC 25922 and *S. aureus* ATCC 25923. The use of 50, 75 and 100 µL hydrogel aliquots corresponded to approximately 12.5, 18.75 and 25 µg AgNPs, respectively, allowing the observed volume-dependent inhibition to be interpreted in relation to nominal AgNP dose. The blank Ch-HI also produced modest inhibition zones, which may reflect the known interaction of cationic chitosan with negatively charged bacterial cell surfaces and subsequent effects on membrane integrity. The larger inhibition zones observed for AgNP-loaded hydrogels suggest an additional contribution from the incorporated phytogenic AgNPs. AgNPs may exert antibacterial effects through membrane disruption, interaction with thiol- and phosphorus-containing biomolecules, oxidative stress generation and interference with bacterial replication, while chitosan may provide complementary membrane-disruptive effects (Huang et al., 2017; Díez-Pascual, 2018; Salleh et al., 2020; Alven and Aderibigbe, 2024).

Recent antimicrobial dressing literature supports this combined-material strategy. Silver-functionalized chitosan hydrogels and AgNP-containing hydrogel systems have been reported to provide silver-associated release, antibacterial effects and improved wound repair in infected or burn wound models. (Chelu et al., 2023; He et al., 2025; Zhao et al., 2025). The present findings align with these reports while adding a comparison of two phytogenic AgNP sources within an injectable thermosensitive chitosan/β-GP hydrogel matrix. However, the antibacterial assessment in the present study was limited to agar diffusion against reference bacterial strains. MIC, MBC, time-kill, biofilm inhibition/eradication assays, clinical or multidrug-resistant isolates, AgNP-only controls and silver sulfadiazine or commercial silver dressing comparators were not included. Therefore, the current data support *in vitro* antibacterial potential under agar diffusion conditions, while further studies are required to define antibacterial potency, killing kinetics, antibiofilm efficacy and clinical relevance.

Cytocompatibility testing in HFF-1 fibroblasts is relevant because fibroblasts contribute to extracellular matrix synthesis, collagen deposition, contraction and tissue remodeling during wound repair. In the present study, cell viability remained above the 70% cytotoxicity threshold after 2 h exposure across the tested 1–50 μg/mL AgNP-equivalent concentration range, suggesting that the formulations were not acutely cytotoxic to fibroblasts under the specific *in vitro* conditions tested (Standardization, 2009). However, this finding should be interpreted cautiously because the exposure duration was short and MTT primarily reflects metabolic activity. Excessive or prolonged silver exposure can impair host-cell function, and cytotoxicity may differ depending on AgNP dose, silver ion release, exposure time and cell type (Haidari et al., 2021a; Pangli et al., 2021). Therefore, future studies should include longer exposure periods, keratinocytes, Live/Dead staining, LDH release and oxidative-stress or inflammatory-marker assays to more fully define the safety profile of these AgNP-loaded hydrogels.

Microbial surveillance showed no detectable culturable bacterial growth from wound swabs during the study period, indicating that the burn wounds remained free from overt infection under the controlled experimental conditions. This is important because it allowed the wound-healing effects of the formulations to be interpreted without the confounding influence of uncontrolled microbial burden. However, the absence of detectable bacterial growth also means that the *in vivo* study primarily demonstrates burn wound repair, local tolerability and regenerative performance, while the antibacterial potential of the formulations is supported by the agar diffusion assay. Future studies using defined infected burn models would be valuable to directly quantify *in vivo* bacterial reduction and confirm antimicrobial efficacy under clinically infected wound conditions (Guo et al., 2024).

Macroscopic wound closure confirmed the functional advantage of incorporating phytogenic AgNPs into the injectable chitosan hydrogel matrix. Rather than acting only as a passive covering, the AgNP-loaded hydrogels appeared to provide a localized antimicrobial and tissue-supportive depot that accelerated the transition from the inflammatory phase toward proliferative wound repair. This interpretation is consistent with recent reports showing that injectable and conformable hydrogel dressings can improve wound-bed contact, maintain hydration and support regeneration in burn and deep-wound models (Cook et al., 2022; Fathi et al., 2024; Li et al., 2025).

The stronger early-to-mid response observed with Ch/Rc-AgNP-HI may reflect a favorable combination of AgNP retention, sustained release, hydrogel swelling and local matrix support during the active proliferative phase of healing. Similar AgNP- or silver-functionalized hydrogel systems have been reported to enhance wound repair by combining antimicrobial activity with controlled silver availability and improved local residence at the wound surface (Haidari et al., 2021a; Haidari et al., 2021b; He et al., 2025; Zhao et al., 2025). In this context, the near-complete macroscopic closure observed in the AgNP-loaded hydrogel groups suggests that the hydrogel platform preserved the biological activity of the phytogenic AgNPs while providing a wound-conformable delivery matrix.

Importantly, accelerated wound closure was not accompanied by increased clinical scores. Daily monitoring showed that all treatment groups remained below the predefined intervention threshold, indicating that repeated intrawound administration of Ch/Rc-AgNP-HI and Ch/Ab-AgNP-HI was not associated with observable systemic distress or adverse clinical signs under the tested conditions. This is important because silver-based wound therapies must balance antimicrobial potency with local and systemic tolerability, particularly when repeated application is required (Pangli et al., 2021; Aldakheel et al., 2023; He et al., 2025). Together with the HFF-1 cytocompatibility data, the clinical score findings support a favourable preliminary safety profile for the AgNP-loaded chitosan hydrogel injections, although further studies assessing silver ion release, local irritation and systemic biodistribution would be required before translational progression.

Histological findings supported the wound closure results. H&E staining indicated improved epidermal organization, reduced inflammatory appearance and more organized tissue architecture in AgNP-loaded hydrogel-treated wounds compared with saline control. Masson trichrome staining further supported improved collagen organization, indicating that the formulations influenced deeper tissue remodeling rather than only superficial wound contraction (Patil et al., 2018; Oryan et al., 2019).

The biochemical findings strengthened this interpretation. Hydroxyproline is widely used as an indirect marker of collagen-associated matrix deposition, while dry matter percentage reflects tissue regeneration and matrix accumulation during repair (Patil et al., 2018; Oryan et al., 2019). Ch/Rc-AgNP-HI produced the highest hydroxyproline and dry matter values at day 14, matching its rapid macroscopic closure at the same time point. Ch/Ab-AgNP-HI showed strong day 21 values, suggesting sustained late-stage matrix maturation. However, the hydroxyproline and dry matter analyses were performed on a limited number of animals at each destructive sampling time point, and the resulting values should be interpreted cautiously alongside the histological and macroscopic findings. In addition, systemic silver exposure was not assessed in this study. Future studies should quantify total silver and ionic silver in blood and major organs such as liver, kidney and spleen using ICP-MS or AAS, particularly after repeated topical AgNP administration, to better define systemic exposure and safety.

Collectively, the macroscopic, histological and biochemical findings support improved wound repair following treatment with the AgNP-loaded hydrogels. However, because murine wound closure is strongly influenced by skin contraction and the histological evaluation was qualitative, these findings should be interpreted cautiously. Future studies incorporating blinded quantitative histomorphometry, re-epithelialisation measurements, granulation tissue thickness, inflammatory cell quantification, angiogenesis markers (e.g., CD31) and cytokine profiling (e.g., IL-1β, TNF-α, IL-6 and TGF-β) will be important to define the underlying repair mechanisms more rigorously. Comparable multifunctional wound hydrogel systems have recently combined injectability, antibacterial activity, controlled release, anti-inflammatory behavior, angiogenic support or collagen-promoting effects to improve repair in complex wounds (Huang et al., 2017; Borhani et al., 2023; Li et al., 2023; Fathi et al., 2024; He et al., 2025). Consistent with this direction, Ahmed et al. (Ahmed et al., 2026) recently reported that pomegranate peel-derived AgNPs incorporated into a hydrogel enhanced wound repair in an *in vivo* full-thickness excision model, particularly when the AgNP hydrogel was pre-exposed to 660 nm red light. That study reported improved wound closure, antibacterial activity against *E. coli* and *S. aureus*, and histological evidence of enhanced angiogenesis, fibroblast proliferation, collagen deposition and reduced inflammation (Ahmed et al., 2026). The present study contributes to this growing area by demonstrating an injectable chitosan/β-GP hydrogel system incorporating two plant-derived AgNP formulations for burn wound repair, without the need for external photoactivation.

Several limitations should be acknowledged. The study used a non-infected small-animal burn model and should be validated further in infected burn wounds, larger wounds and clinically relevant dressing conditions. Silver ion release, total silver quantification, systemic biodistribution and long-term local toxicity were not fully characterized. In addition, cytocompatibility assessment was limited to a short-term 2 h MTT assay using HFF-1 fibroblasts and did not include 24–72 h exposure, keratinocyte assays, Live/Dead staining, LDH release, oxidative-stress markers, inflammatory-marker analysis or full presentation of positive-control cytotoxicity data. Future work should include these studies, together with histomorphometric scoring and bacterial burden analysis in infected wound models, to more comprehensively evaluate safety and translational potential (Chelu et al., 2023; Guo et al., 2024). Nevertheless, the combined formulation, *in vitro* antibacterial, preliminary cytocompatibility and non-infected burn wound repair results provide proof-of-concept for phytogenic AgNP-loaded injectable chitosan/β-GP hydrogels as local wound repair platforms with *in vitro* antibacterial potential (Wang et al., 2021; Zhao et al., 2021; Xie et al., 2023; Zhong et al., 2024).

## 5 Conclusion

Injectable thermosensitive chitosan/glycerol-2-phosphate hydrogel formulations loaded with Rc-AgNPs or Ab-AgNPs were successfully prepared. The hydrogels showed near-physiological pH, temperature-responsive sol-gel transition, AgNP content with high entrapment efficiency, favorable gel fraction and rheological behavior, temperature- and pH-responsive swelling, apparent release of Ag-containing species over 30 h, gradual erosion over 21 days, freeze-dried porous-like morphology, comparative thermal stability, *in vitro* antibacterial activity against *E. coli* and *S. aureus*, and acceptable short-term HFF-1 fibroblast compatibility in a 2 h MTT screening assay.

In a female BALB/c mouse second-degree burn wound model, 50 µL AgNP-loaded hydrogel treatment, corresponding to a nominal AgNP input dose of approximately 12.5 µg per wound, promoted macroscopic wound closure and supported histological and biochemical indicators of tissue repair compared with saline control and blank chitosan hydrogel. Ch/Rc-AgNP-HI showed stronger early-to-mid wound closure trends, while both AgNP-loaded formulations achieved complete wound closure by day 21. Microbial surveillance showed no detectable bacterial growth from wound swabs in any group; therefore, the *in vivo* findings should be interpreted as evidence of improved repair in a non-infected burn wound model rather than therapeutic antibacterial efficacy in an infected wound setting.

Overall, this study demonstrates that two previously characterized phytogenic AgNP systems can be incorporated into injectable chitosan/β-GP thermosensitive hydrogels and evaluated as local burn wound repair platforms with *in vitro* antibacterial potential. Future studies using infected burn wound models, clinically relevant silver comparators, longer cytocompatibility assays, quantitative histomorphometry and silver biodistribution analysis are required to further define antibacterial efficacy, safety and translational potential.

## Data Availability

The original contributions presented in the study are included in the article/supplementary material. Further inquiries can be directed to the corresponding author.
